# A feasibility randomized controlled trial of a community-level physical activity strategy for older adults with motoric cognitive risk syndrome

**DOI:** 10.3389/fragi.2024.1329177

**Published:** 2024-08-08

**Authors:** Shannon M. Hernon, Yashi Singh, Nathan Ward, Arthur F. Kramer, Thomas G. Travison, Joe Verghese, Roger A. Fielding, Christopher Kowaleski, Kieran F. Reid

**Affiliations:** ^1^ Laboratory of Exercise Physiology and Physical Performance, Brigham and Women’s Hospital, Harvard Medical School, Boston, MA, United States; ^2^ Department of Psychology, Tufts University, Medford, MA, United States; ^3^ Department of Psychology, Center for Cognitive and Brain Health, Northeastern University, Boston, MA, United States; ^4^ Marcus Institute for Aging Research, Hebrew Senior Life, Boston, MA, United States; ^5^ Division of Gerontology, Beth Israel Deaconess Medical Center, Harvard Medical School, Boston, MA, United States; ^6^ Department of Neurology, Albert Einstein College of Medicine, Bronx, NY, United States; ^7^ Department of Medicine, Albert Einstein College of Medicine, Institute of Aging Research, Bronx, NY, United States; ^8^ Nutrition, Exercise Physiology and Sarcopenia Laboratory, Jean Mayer USDA Human Nutrition Research Center on Aging, Tufts University, Boston, MA, United States; ^9^ City of Somerville Council on Aging, Health and Human Services Department, Somerville, MA, United States

**Keywords:** motoric cognitive risk syndrome, physical activity, community-based, feasibility, pre-dementia

## Abstract

The motoric cognitive risk syndrome (MCR) is a syndrome characterized by subjective memory complaints and slow walking speeds that can identify older adults at increased risk for developing Alzheimer’s disease or a related dementia (ADRD). To date, the feasibility of community-based physical activity (PA) programs for improving outcomes in MCR have yet to be examined. To address this knowledge gap, we conducted a translational randomized controlled trial (RCT) comparing 24-weeks of PA to a healthy aging education (HE) control intervention delivered within the infrastructure of an urban senior center in Greater Boston (clincaltrials.gov identifier: NCT03750682). An existing senior center employee was trained to administer the multimodal group-based PA program that included moderate-intensity aerobic walking, strength, flexibility and balance training. A total of 79 older adults attended the senior center for a screening visit, of whom 29 met the MCR criteria and 25 were randomized to PA or HE (mean age: 74.4 ± 7 years; BMI: 32.4 ± 7 kg/m^2^; 85% female; 3MSE score: 92.4 ± 7; gait speed: 0.52 ± 0.1 m/s; SPPB score 4.8 ± 1.9). Due to the Covid-19 pandemic the study was stopped prematurely. Participants could successfully adhere to the study interventions (overall attendance rate: PA: 69% vs. HE:70% at study termination). Participants also successfully completed baseline and follow-up study assessments that included a computerized cognitive testing battery and objective tests of physical performance and functional exercise capacity. No study-related adverse events occurred. Notable trends for improved cognitive performance, gait speed and 6-min walk distance were exhibited in PA compared to HE. Our study provides important preliminary information to aid the design of larger-scale RCTs of PA that may help to preserve the independence of vulnerable older adults at high risk for ADRD in community-based settings.

## Introduction

The development of Alzheimer’s disease or a related dementia (ADRD) has a profound impact on the daily functioning of older adults, their families, and healthcare systems. Currently, over 50 million people worldwide are living with ADRD and its prevalence is estimated to triple by 2050 (Scheltens et al., 2021). Therefore, the development and widespread implementation of more effective lifestyle strategies to prevent, delay, or halt the onset of ADRD, particularly among high-risk older adults, is an urgent public health priority.

Physical activity (PA) remains one of the most practical and effective lifestyle interventions for preserving cognition and brain health in older adults. A number of mechanistic studies and clinical trials have demonstrated the protective effects of PA on brain health which include increased brain angiogenesis, cerebral blood flow, hippocampal volume, neurogenesis and decreased neuroinflammation (Colcombe and Kramer, 2003; Kramer et al., 2005; Colcombe et al., 2006; Erickson et al., 2011; Voss et al., 2013). Despite this well-established evidence, efforts to translate the beneficial effects of PA interventions to mitigate the development of ADRD, particularly in high-risk older adults, outside of laboratory or clinical settings, are lacking.

To address this translational research gap, we conducted the Engage for Brain Health (ENGAGE-B) study. We sought to determine the feasibility of a community-level PA intervention for older persons who may be at high-risk for developing ADRD by specifically targeting older adults with motoric cognitive risk syndrome (MCR) in a community-based setting. MCR is a pre-dementia syndrome characterized by objectively measured slow gait and subjective cognitive complaints (Verghese et al., 2013). Identification of older adults with MCR does not require resource-intensive, clinic-based neuropsychological testing or brain imaging, yet its assessment can identify older persons who are at substantially greater risk of developing subsequent ADRD (Verghese et al., 2013; Verghese et al., 2014). Despite the utility of MCR assessment for identifying high-risk older adults in community settings, no study to date has attempted to implement and study the effects of a PA intervention among this high ADRD-risk phenotype. Such a strategy could provide an earlier window of opportunity for preventing or delaying ADRD among this distinctly vulnerable population of older adults.

ENGAGE-B had several overarching aims. First, we tested the feasibility of identifying and recruiting older adults with MCR for a randomized controlled trial (RCT) of PA or a control intervention of healthy aging education (HE) within a large urban senior center setting in Greater Boston, MA, United States. As there have been no previous RCTs of lifestyle interventions among older adults with MCR, our primary feasibility objective was to establish screening, eligibility and enrollment yields from a community setting that would likely be informative for the design of a future, larger scale community based RCT in MCR. Second, we examined the feasibility of training a current senior-center-based employee to administer a structured multimodal PA program (consisting of moderate-intensity walking, strength, flexibility and balance training) and the ability of older adults with MCR to safely adhere to PA within this community setting. The objective of this training was to efficiently train and certify this individual on all major aspects of the PA intervention in a single day. If successful, this would represent a scalable approach for designing a larger RCT whereby multiple community-based employees could be identified and similarly trained. For our PA safety objective, we sought to demonstrate that there would be no increased rate of study-related AEs or SAEs in PA relative to HE. For PA adherence, we considered an overall PA session attendance rate of ≥60% successful. This adherence rate was based on our prior experience with community-based PA studies while also recognizing that an MCR older adult population would likely have many physical impairments and co-existing chronic medical conditions that could potentially limit PA adherence. Lastly, and to further aid the design of future RCTs and community-based studies in MCR, we deemed it important to demonstrate that we could successfully attain valid tablet-based measures of cognitive performance and other meaningful assessments that included measures of physical performance, dual task walking, exercise capacity and self-reported health from MCR older adults in this community setting.

## Methods

### Study design and location

We performed a parallel-group, single-blind, community-based RCT to examine the feasibility of 24 weeks of physical activity (PA) versus a healthy aging education (HE) control intervention in older adults with MCR (clinicaltrials.gov identifier: NCT03750682). The study was conducted in a large urban senior center in the Greater Boston area through an established community outreach partnership with a local council on aging (Somerville Council on Aging, Somerville, MA, United States). Approximately 100 older adults typically utilize the senior center daily and the council on aging serves over 5,500 older adults in the wider Somerville, MA, community through provision of transportation and access to wellness and educational activities, socialization opportunities, and meals programs.

### Participant recruitment

Study recruitment was initiated in February 2019 and participants were recruited through social media postings, advertisements, newsletters, distribution of study flyers and targeted community outreach conducted by the study investigators in collaboration with senior center staff. Older adults who were already attending the senior center for services were initially targeted for participation. Specific outreach activities were subsequently performed which primarily consisted of senior center staff and study team personnel visiting senior housing and assisted living facilities that were supported by the Somerville Council on Aging. During these events, study information was provided to potential participants. Participants who were initially interested in the study were pre-screened via telephone or in person and were considered eligible for an in-person screening visit if they were 60–89 years, community-dwelling (including senior housing and assisted living facilities), sedentary (not performing any structured physical activity within the past 6 months), ambulatory, reported a subjective memory complaint and were willing to be randomized to the physical activity or healthy aging education interventions. Eligible participants were then invited to participate in additional senior-center based screening procedures and considered to have MCR if they met all of the following criteria: 1. self-reported memory complaint assessed using question 10 from the Geriatric Depression Scale (Yesavage et al., 1982); 2. slow gait speed objectively measured during the 4-m test from the short physical performance battery (SPPB) test (Guralnik et al., 1994) which we defined as gait speed below based on previously described age-appropriate mean values (age 60–74 years: <0.70 m per second and age 75+: <0.60 m per second) (Verghese et al., 2013; Verghese et al., 2014); 3. absence of mobility-disability (inability to ambulate even with assistance or walking aids) 4. absence of dementia diagnosis. Each participant’s primary care physician (PCP) confirmed the absence of a prior diagnosis of dementia. Participants also completed a medical history questionnaire and were excluded if they had an acute or terminal illness, significant cognitive impairment (Modified Mini-Mental State Examination Score (3MSE) < 80) (Teng and Chui, 1987), myocardial infarction or upper and lower extremity fracture in the previous 6 months, symptomatic coronary artery disease or uncontrolled hypertension. Participants who met the study entry criteria and were given medical clearance to participate by their PCP were deemed eligible and were randomized to either intervention group. Signed informed consent was obtained from all study participants and this study was approved by the Tufts University Health Sciences Institutional Review Board.

### Intervention

We trained an existing senior center-based employee to serve as the community health promoter of multimodal PA (PA-CHAMP). The PA-CHAMP was the senior center health and wellness coordinator, had a college level of education (bachelor’s degree), was certified in cardiopulmonary resuscitation (CPR) and use of an automated external defibrillator (AED) certified, and had prior experience in exercise programming for older adults. Initially, the senior center leadership had suggested an outreach coordinator as the individual who would serve as the PA-CHAMP. This individual had a college level of education (associate’s degree), no background in exercise programming for older adults, but was enthusiastic for the role. However, after learning more about the training procedures and the necessary commitment to serve as the PA-CHAMP, this individual declined the role citing competing job responsibilities as the major limiting factor.

The interactive training and certification of the PA-CHAMP was conducted over the course of a single day. Previous studies have used a variety of approaches to train community-based staff to deliver PA interventions, including multiple day training workshops and experiential training through active program participation (Brach et al., 2018; Stathi et al., 2018; Wert et al., 2018). We decided to limit our initial training to a single day to assess the practical scalability of our training approach. The didactic training started with an initial presentation by the study PI on the design and rationale for the group-based multimodal intervention and a review of videos of all exercises. Other key elements reviewed included the monitoring of exercise intensity using the Borg rate of perceived exertion (RPE) scale (Borg, 1970), principles of PA progression, PA safety, and a process for resumption of PA after a medical illness or event. The PA-CHAMP was also trained to monitor potential adverse experiences and symptoms before, during and after exercise, including syncope, chest pain and abnormal vital signs and the importance and proper method of warming-up prior to and cooling down following PA. The training then went on to include PA practice and demonstration sessions with study staff and review of the PA manual and PA tip-sheet (see Supplementary Material) before final certification by the PI. An abbreviated version of the PA-CHAMP training presentation is also provided as a Supplementary Material. The full PowerPoint presentation of the training, which has embedded videos of all training components, may be requested from the corresponding author.

Participants randomized to the 24-week PA intervention were required to attend twice weekly group -based sessions (up to six participants per session) which each lasted 45–60 min in duration. Sessions were led by the PA-CHAMP and consisted of a structured regimen of progressive, moderate-intensity aerobic walking exercise, strength, flexibility and balance training, which was adapted from our prior studies (Fielding et al., 2011; Rejeski et al., 2013; Laussen et al., 2016; Reid et al., 2019). The PA sessions were individualized and progressive towards a goal of ∼25–30 min of aerobic walking, ∼10 min of lower extremity strength training using ankle weights (2 sets of 10 repetitions), a short regimen of flexibility exercises (∼5 min), followed by ∼10 min of balance training. The participants began with lighter intensity and gradually increased intensity over the first 2–3 weeks of the intervention (see PA manual in Supplementary Material). The PA-CHAMP delivered the program within the existing infrastructure of the senior center with the corridors used to facilitate the aerobic walking exercise and a large dining room with an existing supply of chairs and ankle weights used to facilitate the muscle strengthening, flexibility, and balance training components. During all sessions, an AED and other CPR-AED on-site trained staff were available in case of a medical emergency.

### Control

The 24-week HE control intervention was also administered in a group format by a study staff member within a private classroom at the senior center. Each session lasted approximately 45–60 min in duration and consisted of biweekly seminars, presentations and workshops on topics of relevance and anticipated interest to older adults. Examples of these topics included nutrition for healthy aging, dental health for older adults, stress management, sleep quality and fall prevention. Recommendations for physical activity were not discussed as part of the HE curriculum. The format, duration and content of the HE intervention was consistent with previous studies (Fielding et al., 2011; Reid et al., 2019).

### Intervention fidelity, adherence and safety

To maintain fidelity, intervention sessions were scheduled on days and times that minimized potential interference from other daily events and activities at the senior center and the HE intervention was scheduled on an alternate weekday to the PA sessions. Participants were also requested not to discuss their intervention assignment at the senior center throughout the duration of the study. To ensure fidelity of PA, the study PI regularly attended and observed PA sessions and provided feedback to the PA-CHAMP. The frequency of these visits was at least weekly for approximately the first 2 months of the study and thereafter bimonthly or as necessary until study cessation. Intervention adherence in both arms was defined as the percentage of sessions attended relative to the total number of possible sessions, excluding nonattendance for medical/illness-related reasons. Throughout the course of the intervention, the PA-CHAMP was provided with regular guidance on how to monitor and address adherence problem solving strategies with study personnel. The PA-CHAMP briefly probed and monitored for the occurrence of potential AEs and symptoms before and during each PA and during follow-up telephone calls. In addition, study staff also administered a questionnaire on a biweekly basis to monitor the occurrence of any additional AEs. Participants were also monitored for the occurrence of adverse events during the study by the study assessor. The study physician adjudicated whether AEs were related to either intervention. Nonserious AEs were defined as conditions that may have been unpleasant and bothersome to the participant but did not require discontinuation of participation. AEs were considered serious if they involved death, hospital admission, or the occurrence of a persistent or significant disability/incapacity.

### Outcome assessments

The study assessments were conducted by a blinded assessor before randomization and repeated at week 24. An additional interim measure of cognitive performance was performed at week 12. The cognitive testing battery of eight standard cognitive tasks was conducted on an iPad tablet device using the mobile application *BrainBaseline* as previously described (Lee et al., 2012; White et al., 2020; Ward et al., 2021). The cognitive tasks consisted of a digit symbol substitution task, a digit span task, an n-Back task, a processing speed task, an Erikson flanker task, a Stroop task, a task switching task, and a trail making task, which have been validated in prior research (Lee et al., 2012). To further aid generalizability, these individual tasks were combined into several different composite scores of working memory, inhibitory control, task-shifting, processing speed, executive function and an overall cognition composite measure. The working memory composite consisted of the digit span and N-Back tasks; the inhibitory control composite consisted of the Erikson flanker and Stroop tasks; the task-shifting composite consisted of the task switching and trail making tasks; the processing speed composite consisted of the digit symbol substitution and processing speed tasks; and the overall cognition composite was computed by combining all of the eight individual tasks and the 3MSE measure. To create the composite scores, z-scores were first calculated for the individual cognitive variables with the Stroop, Erikson Flanker, processing speed, task-switching and trails tasks being multiplied by −1 so that for all measures better performance was associated with larger values and poorer performance associated with smaller values. The dual task walking test was performed with participants who were first asked to walk 7 m at normal pace, without any other simultaneous task. Next participants were given a letter (e.g., S, T, or M depending on the day of the month they were born) and asked to name as many animals as they could think of whose name started with that letter while walking the same 7-m distance at their normal walking pace (i.e., Dual Task) (Ward et al., 2021). Other study assessments included the 6 min walk test (Laboratories, 2002), nondominant handgrip strength using an adjustable, hydraulic dynamometer (JAMAR 5030JI, Bolingbrook, IL) and self-reported fear of falling (Yardley et al., 2005).

### Statistical analysis

Consistent with sample sizes of previous preliminary studies, we planned a sample size of approximately 40 total participants to meet our study feasibility aims. All statistical procedures were performed using SPSS statistical software (Version 28). Descriptive statistics were used to summarize baseline characteristics, and the feasibility aims of participant recruitment, intervention adherence, safety and assessment completion rates. Descriptive statistics were also used to summarize the baseline, week 12 and week 24 study assessment data among participants who attended at least 50% of scheduled intervention sessions. Due to the small sample sizes and considering that our study was unable to reach target enrollment due to early termination, statistical power for formal inference was lacking, and therefore no hypothesis tests were conducted.

## Results

### Recruitment and baseline participant characteristics

**FIGURE 1:**
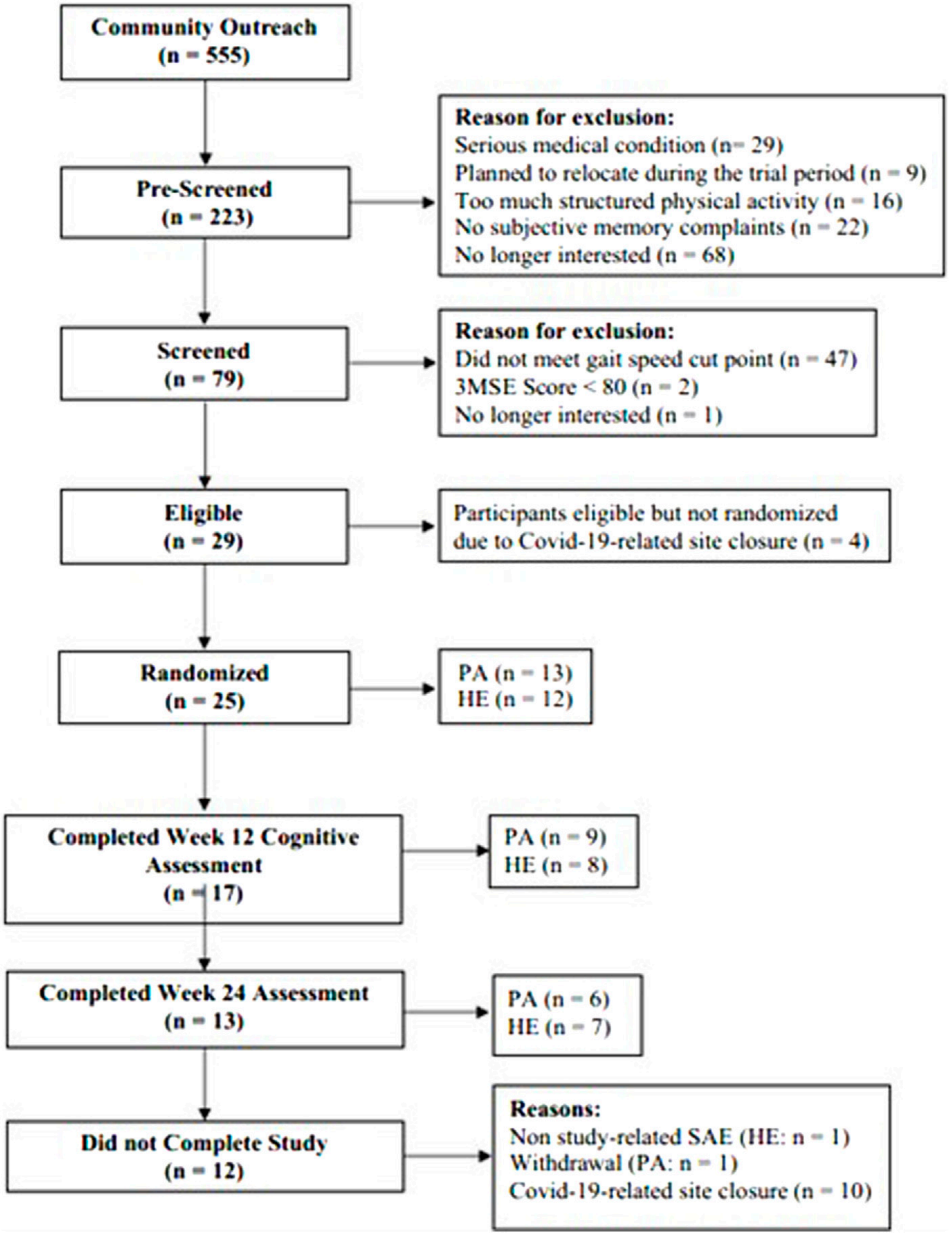
CONSORT diagram of participant flow through screening and trial procedures.

Figure 1 describes the participant recruitment flow throughout the study. A total of 555 individuals responded to recruitment and outreach efforts. Of these, 223 individuals were prescreened to determine eligibility for in-person screening at the senior center. Of these prescreened participants, 114 were excluded as ∼10% did not have a subjective memory complaint, ∼13% had a serious medical condition, ∼7% were too physically active, ∼4% planned to relocate during the trial period, and ∼31% were no longer interested after completing the pre-screening interview. Seventy-nine of the pre-screened participants (∼35%) attended a screening visit of whom 29 participants were determined to be eligible, representing a prescreening to eligibility yield of 13%. A total of 25 participants were randomized to PA (*n* = 13) or HE (*n* = 12) representing a screening to randomization yield of 32%. Of the randomized participants, 16 (64%) were independently living in the community and 9 (36%) resided in senior housing facilities. Twelve (48%) of the randomized participants were regularly attending the senior center while the remaining 13 (52%) were residing within the community and agreed to come to the senior center to participate in the study. The majority of randomized participants (48%) were recruited through direct outreach by senior center staff; 32% were recruited from informational events that were held in senior housing facilities, and the remaining 20% were respondents to a flyer or social media posting about the study. Four additional participants were eligible but did not advance to randomization prior to the study’s early termination, which was due to closure of the senior center because of the Covid-19 pandemic, and IRB directives to cease all intervention related activities involving older adults. At study cessation, 10 participants (6 PA and 4 HE) participants were actively participating in various stages of their respective interventions (PA: week 7: n = 1; weeks 10–14: n = 5; HE: weeks 8–10: n = 3, week 20: n = 1). A total of 13 participants fully completed the study interventions and week 24 assessments.

Baseline descriptive statistics are presented in Table 1. Most participants were female (80%), 28% were minorities and there was a high burden of chronic medical comorbidities. On average, our MCR sample had global cognitive deficits (3MSE), major gait impairments, severe mobility limitations and low functional exercise capacity as determined from their 6MWT performance.

**TABLE 1:** Baseline characteristics of randomized MCR participants.

Baseline Characteristics (n = 25)	Mean ± SD [n (%)]	(Min -Max)
Age, years	74.4 ± 6.8	(65–89)
BMI, kg/m^2^	32.4 ± 7.0	(19.6–44.3)
Medications, n Medical Conditions, n	6.4 ± 4.5 4.3 ± 2.6	(0–20)
Sex		
Female	20 (80%)	
Male	5 (20%)	
Race		
White	18 (72%)	
African American	4 (16%)	
Asian	2 (8%)	
Hispanic/Latino	1 (4%)	
Education		
High School	11 (44%)	
College	14 (56%)	
3MSE Score	92.4 ± 6.7	(80–100)
SPPB Score	4.8 ± 1.9	(1–8)
Gait Speed, m/s	0.52 ± 0.11	(0.17–0.69)
6MWT, (m)	234 ± 74	(50–378)

Values are presented as mean ± SD [n (%)]. BMI, body mass index; 3MSE, Modified Mini Mental Status Exam; SPPB, short physical performance battery; m/s, meters per second; 6MWT, 6-min walk test; m, meters.

### Intervention safety and adherence

No on-site or study-related serious adverse events (SAEs) occurred in the PA of HE intervention groups. Non-study-related SAE’s did occur outside of the intervention activities in 1 PA participant (myocardial infarction) and 2 HE participants (pneumonia; acute coronary syndrome). One of these HE participants discontinued their study participation as a result. No study-related adverse events (AEs) occurred in either intervention arm. Three non-study-related AEs occurred in 1 PA participant (musculoskeletal) and 1 HE participant (musculoskeletal and dermatologic). One participant completed baseline assessments and was randomized to PA but elected not to participate before attending for their first PA session. This participant was withdrawn from the trial by the study investigators. The average adherence rates, including the participants who were active in the study at the time of study cessation but excluding intervention visits missed due to medical reasons, were 69% in PA and 70.0% in HE. Unadjusted adherence rates were 62% in PA and 64% in HE.

### Study assessments

#### Measurement

All eligible participants could fully complete all baseline testing procedures that included the cognitive assessment battery, objective tests of physical performance, dual-task walking, functional exercise capacity and other self-report measures.

#### Preliminary findings

**FIGURE 2:**
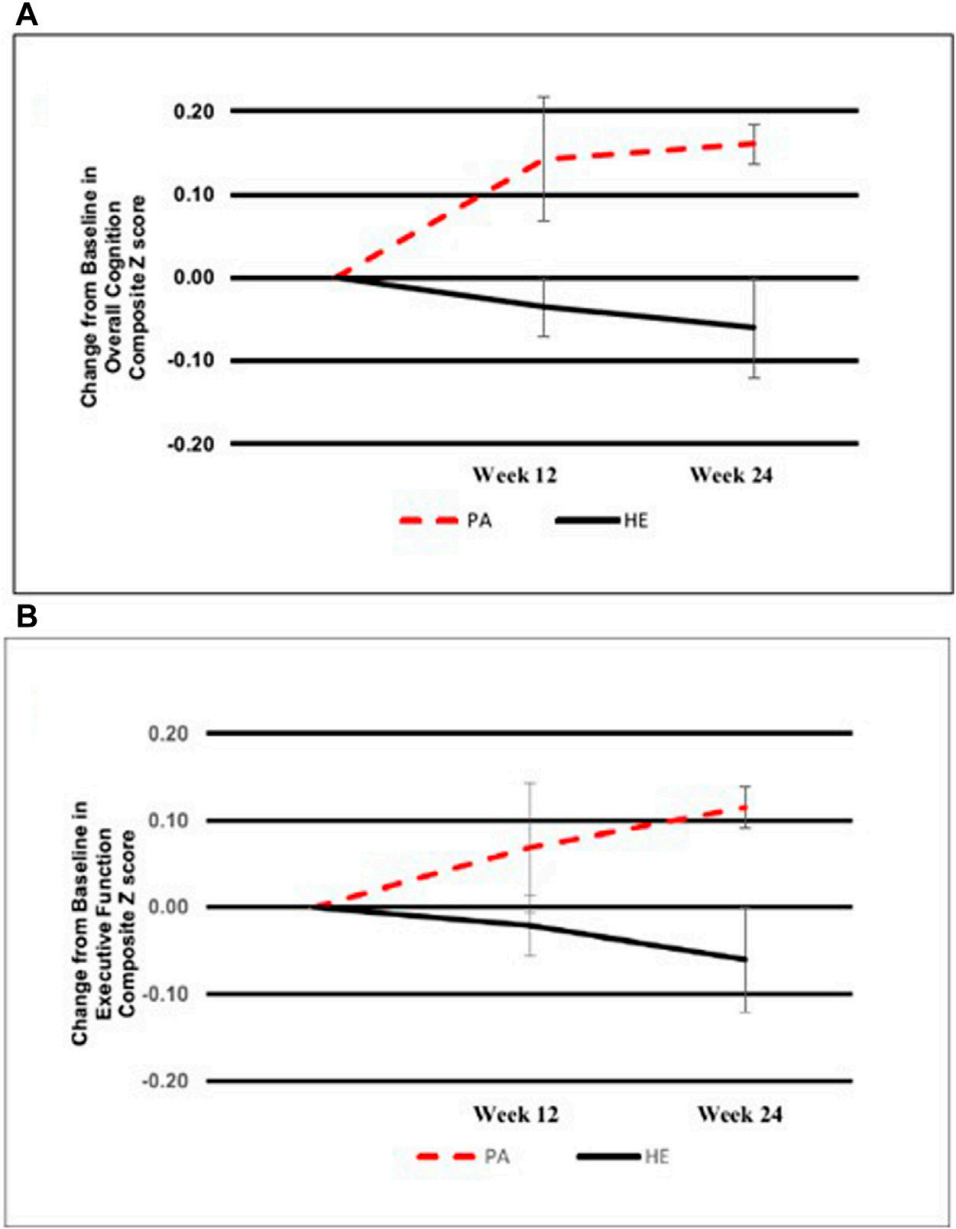
Effects of physical activity and healthy aging education on overall cognition and executive function in older adults with motoric cognitive risk syndrome. Participants who completed the PA and HE interventions (with > 50% adherence) and both of the week 12 and week 24 computerized cognitive battery assessments. Despite the small sample sizes (PA: n = 5 vs. HE: n = 7), at both timepoints there was a notable trend for improvement in overall cognition composite scores **(A)** and executive function composite scores **(B)** in PA compared to HE. Values are presented as mean change ± SE.

The data provided limited evidence suggesting meaningful improvements in cognitive performance in PA beyond those observed in HE as expressed among participants with ≥50% intervention adherence (Figure 2). Changes in the overall cognitive composite score were mirrored by similar changes in executive function. Trends for improvements in task-shifting and the processing speed cognitive domain composites were observed in PA vs. HE but not for working memory or inhibitory control composite scores (Supplementary Figure S1). Table 2 displays the results of additional study assessments. The pattern of greater improvement in cognitive function attending assignment to PA relative to HE was mirrored by similar differences in improvements in SPPB score, gait speed, dual task gait, and 6MWT distance. Three PA participants (60% of completers) had gait speed improvements that resulted in them not being considered as having MCR based on baseline MCR gait speed cut scores.

**TABLE 2:** Baseline, week 24 and change values for study assessments after PA and HE.

	PA (n = 5)			HE (n = 7)		
	Baseline	Week 24	Δ	Baseline	Week 24	Δ
SPPB Score	5.8 ± 1.3	6.6 ± 1.9	0.8 ± 1.6	4.4 ± 2.4	3.7 ± 1.3	−0.7 ± 2.0
4 m Gait Speed (m/s)	0.56 ± 0.08	0.71 ± 0.13	0.14 ± 0.17	0.52 ± 0.10	0.58 ± 0.08	0.06 ± 0.05
Six Minute Walk Distance (m)	262.1 ± 88.8	283.8 ± 68.3	21.6 ± 38.3	231.5 ± 52.4	203.2 ± 76.7	−28.3 ± 45.9
Gait Speed during Six Minute Walk (m/s)	0.73 ± 0.24	0.78 ± 0.19	0.05 ± 0.10	0.64 ± 0.14	0.59 ± 0.19	−0.05 ± 0.11
Gait Speed During 7 m Dual Task (m/s)	0.48 ± 0.14	0.64 ± 0.18	0.16 ± 0.25	0.45 ± 0.08	0.50 ± 0.16	0.04 ± 0.16
Handgrip Strength (kg)	11.6 ± 4.8	11.8 ± 2.7	0.1 ± 2.8	15.5 ± 5.0	17.7 ± 5.8	2.2 ± 6.2
Geriatric Depression Scale	1.6 ± 0.5	2.8 ± 1.3	1.2 ± 1.5	2.9 ± 2.6	4.6 ± 2.0	1.7 ± 1.9
FESI	32.8 ± 14.9	29.8 ± 16.1	−3.0 ± 10.7	27.1 ± 8.2	34.7 ± 9.8	7.6 ± 8.3

Values are presented as mean ± SD. Δ, change from baseline to week 24; SPPB, short physical performance battery test; m, meters; m/s, meter per second; kg, kilograms; FESI, Falls Efficacy Scale International.

## Discussion

ENGAGE-B has demonstrated the preliminary feasibility of a pragmatic community-based PA intervention for older adults with MCR. We successfully recruited older adults with MCR from outside of a clinical setting for an RCT and our findings show that these high ADRD-risk older adults could safely and appropriately adhere to a relatively long-term intervention of multimodal PA. Our translational approach also demonstrated the feasibility of training an existing community-based employee (PA-CHAMP) to administer PA which may represent a highly scalable approach for future intervention implementation. We also report that MCR participants could successfully complete multiple assessments that included a computerized cognitive assessment battery, measures of physical performance and functional exercise capacity outside of a laboratory or clinical research setting. Although our small sample size limits the interpretation of our intervention effects, which should be further verified in an appropriately designed efficacy study in MCR, our results are encouraging as many of the observed changes in our study assessments after PA may be considered meaningful improvements when compared to prior studies of these endpoints in older adults with cognitive and mobility-related impairments (Perera et al., 2006; Hindin and Zelinski, 2012; Chen et al., 2020; McDermott et al., 2021).

The MCR phenotype was first described in 2013 (Verghese et al., 2013). To our knowledge, ENGAGE-B represents the first RCT to examine the feasibility or preliminary effects of PA or any other lifestyle intervention in MCR. Our investigation has revealed several important considerations to aid the design of future and larger-scale RCTs for MCR. We have demonstrated that a highly vulnerable MCR phenotype can be successfully recruited from the community and can appropriately adhere to an intervention of relatively long-term duration (24 weeks). Our observed recruitment yields reaffirm that it is feasible to recruit older adults for an RCT from more general non-clinical community-based settings. Indeed, while our MCR sample had a high burden of chronic medical conditions, severe mobility and gait limitations, cognitive deficits and low functional exercise capacity, participants could successfully adhere to our 2 × week group-based PA intervention at the senior center. The observed PA adherence rates (∼70%) were consistent with rates observed in large-scale clinical trials in older adults that had substantial resources for optimizing adherence and retention in vulnerable older adults (Pahor et al., 2014; Sink et al., 2015). We believe that key factors for our strong adherence rates included the group-based PA format, having PA delivered by a PA-CHAMP who was an existing and familiar senior center staff member, and having PA embedded within the existing environment of the senior center as many of these older adults were already regularly attending the center for congregate meals, social activities and other services. Transportation was also provided to all participants as part of a broader meals program offered by the senior center, which we believe eliminated a major participation barrier and contributed to our strong adherence rates across both intervention groups. Our findings highlight the potential strength of our strategy of harnessing the existing environment, infrastructure, and personnel of a community-based setting to implement a PA intervention in high ADRD-risk older adults. However, it is important to note that our approach and findings may not be entirely generalizable to other community-based senior centers. Our PA intervention was performed entirely indoors, largely due to the local built environment and the variability of the climate in Boston, United States. It may be plausible that future interventions that incorporate more outdoor-based activities could have alternative or additive benefits in MCR. We also believe the provision of transportation was critical to the successful implementation of the current study and an important factor to consider for the design of future trials of senior center-based PA where transportation is not readily available to potential participants. Another key consideration for future studies concerns the selection of the PA-CHAMP. Our initial plan for the senior center outreach coordinator to serve as the PA-CHAMP did not come to fruition as this individual, although enthusiastic, had too many other competing job responsibilities. This was an important lesson learned and highlights the need to allocate sufficient resources to community agencies for staffing to lead the implementation of interventions. Based on our experience in ENGAGE-B, these individuals are critical to the success of any such endeavor. However, it may be important to note that while the selection of existing community-based staff may offer a more pragmatic approach for the delivery of community-level interventions, a prior study has reported reduced effectiveness of physical activity intervention delivered by community-based staff when compared with intervention delivery by non-community-based staff with professional expertise in exercise physiology and physical therapy (Brach et al., 2018). Future studies should further examine the optimal approaches for delivery of community-level PA interventions in MCR.

The definition of MCR used in this study was based on previously established cognitive complaint criterion and objective assessment of gait speed (Verghese et al., 2013; Verghese et al., 2014; Ward et al., 2021). For our clinical trial enrollment criteria, we further operationalized the gait speed cut scores based on the previously published age and sex-specific values and used more global age adjusted cut scores without adjusting for sex. A high proportion of participants who attended the senior center for a screening visit did not meet our gait speed eligibility criteria (∼60%). This screen failure rate is substantially higher than previous studies of group-based exercise in community setting that did not have strict inclusion criteria based on low gait speed or older adults with concomitant impairments in gait and cognition (Brach et al., 2017; Reid et al., 2019). This observation highlights the challenges of recruiting a dual gait and cognitive decline phenotype from the community, and such knowledge may be important for the design of future studies in MCR, particularly with respect to the optimization of recruitment efforts and potential minimization of participant burden. Future clinical trials should also consider the appropriate selection of MCR diagnostic criteria including both age and sex adjusted cut scores while also considering the use of more comprehensive cognitive complaint inventories or recently developed screening questionnaires that may lend greater precision to the community-based assessment of MCR and the identification of ADRD-risk older adults (Ayers et al., 2022).

Aside from the early termination of the RCT, the trial had other limitations as designed. Our PA-CHAMP had prior experience in delivering exercise training among older adults, which may limit the generalizability of our community-level strategy to other settings with personnel without this prior experience. Our PA intervention fidelity monitoring was subjective and not based on established fidelity monitoring frameworks. Aligning our work with these frameworks could have further enhanced both the implementation of PA and the knowledge gained from our study (Bellg et al., 2004; Cross et al., 2022). Although the vast majority of participants received transportation to and from the senior center, the PA group had a higher frequency of intervention sessions relative to the HE group. The additional scheduling, planning and travel to and from the senior center for the PA intervention may have provided an additional stimulus to participants and a potential confounding factor for our observed effects. Another important limitation of our study was a lack of qualitative information on participant’s preferences or satisfaction with the PA intervention or other qualitive assessment of social engagement or PA participation post study. Such information may have been very informative and would have complemented our quantitative study findings. Finally, we chose multimodal PA as the active intervention in ENGAGE-B as we maintain that it currently represents one of the most effective yet scalable lifestyle intervention for potentially improving outcomes in MCR and ADRD-risk. It remains unknown as to what the optimal PA intervention or the relative importance of prioritizing the improvement of cognitive or motoric deficits on MCR outcomes. It is possible that our PA intervention, which has multiple training components within the same intervention (e.g., aerobic, strength, balance, flexibility training) more simultaneously targets the dual decline of cognitive and motor performance and associated multifactorial pathophysiology in MCR. However, other lifestyle interventions or combination of interventions, that have more cognitively engaging or synergistic intervention ingredients may be a more effective interventions and warrant investigation in MCR (Ambrose et al., 2021; Baker et al., 2023).

Despite these aforementioned limitations, our study has many positive findings, and we present a promising approach for the development of an efficacious lifestyle intervention in MCR that should be further tested in larger and appropriately powered studies. As earlier intervention provides a greater opportunity to prevent, halt or reverse the debilitating trajectory of ADRD, establishing a pragmatic lifestyle intervention in older adults at high risk for ADRD has major public health significance. For example, based on simulation models, a therapeutic intervention implemented among high ADRD-risk older adults in 2025, that could delay the onset of ADRD for at least 5 years, would result in a 41% lower ADRD prevalence rate by 2060 and a projected reduction in overall societal costs of approximately 40% (Zissimopoulos et al., 2014).

In conclusion, ENGAGE-B has demonstrated the initial feasibility of a community-level, multimodal PA intervention in older adults with MCR. Additional larger scale RCTs are necessary to further develop and test the efficacy of our community-based approach for minimizing, halting or reversing cognitive decline in MCR. Such research could ultimately lead to findings that could have significant, expeditious and widespread impact on the quality of life of expanding populations of vulnerable older adults at high risk for ADRD, their caregivers, families, and public health systems.

## Data Availability

The raw data supporting the conclusions of this article will be made available by the authors, without undue reservation.

## Supplementary material

SUPPLEMENTARY FIGURE S1 Effects of physical activity and healthy aging education on other domains of cognitive performance in older adults with motoric cognitive risk syndrome. Participants who completed the PA and HE interventions (with > 50% adherence) and both of the week 12 and week 24 computerized cognitive battery assessments. Trends for improvements in task-shifting **(A)** and the processing speed **(B)** cognitive domain composites were observed in PA vs. HE but not for working memory **(C)** or inhibitory control **(D)** composite scores. Values are presented as mean change ± SE.
