# Supplementary material for: A feasibility randomized controlled trial of a community-level physical activity strategy for older adults with motoric cognitive risk syndrome
Source: Front Aging. 2024 Aug 8;5:1329177. doi: 10.3389/fragi.2024.1329177 (PMC11339030; doi:10.3389/fragi.2024.1329177)
Supplement: Supplementary file 1 [file DataSheet3.docx]

**ENGAGE-B Physical Activity (PA) Intervention**

**PA intervention consists of 4 major components (walking, stretching, strength, balance training). Each session will range from 45-60 mins in duration. For optimal benefits, we think that 2 sessions per week will be most beneficial.**

**Walking Exercise:** is the primary mode of physical activity for ENGAGE-B.

- Designate duration for each participant according to progression (15-30 mins avg.).
- Track time
- Assess mid-walk rate of perceived exertion (RPE) (target RPE = 13, from a 6-20 RPE scale)
  - Warm-up/cool down phase

**Strength Exercises**: 2 sets x 10 reps, approx. 60 sec rest intervals. Target RPE = 15.

*Wide leg squat* – rising in/out of chair

*Knee extension* – with ankle weights, seated, 10 reps one side then repeat with other leg

*Leg curl* – with ankle weights, use chair/wall for balance, alternate right/left leg 10x for one set

*Side hip raise* – with ankle weights, 10 reps one side then repeat with other leg

*Toe stands* – no ankle weights

**Stretching Exercises**: static, hold for 20-30 seconds

*Hamstring/calf* – bend at waist, in front of chair

*Quadriceps* – use wall/chair for balance, may need to hold client’s knee at 90°

*Upper back* – interlock fingers in front of body, roll palms away, reach out

*Chest/shoulders* – interlock fingers behind body, pinch shoulder blades, extend arms

**Balance Exercises:** 4 tiers

Tier 1 -Lateral side step – 5 steps each direction X 3 sets, 2 hand support

-Hip circles – 5 each direction

-Toe stands – hold for 5 seconds x 10, 2 hand support

-One leg stand – hold for 5 seconds x 10 with each leg, alternate legs, 1 hand support

Tier 2 -Lateral side step – 5 steps each direction X 3 sets, 1 hand support

-Toe stands – hold for 5 seconds x 10, 1 hand support

-One leg stand – hold for 5 seconds x 10 with each leg, alternate legs, 2 hand support

-Forward Step – 10 steps each side, alternate legs, with support

-Backward Step – 10 steps each side, alternate legs, with support

Tier 3 -Lateral side step – 5 steps each direction X 3 sets, no hands

-Lateral cross-over step – 5 steps each direction X 3 sets

-Toe stand – hold for 5 seconds x 10, no hands support

-Forward Step – 10 steps each side, alternate legs, no support

-Backward Step – 10 steps each side, alternate legs, no support

Tier 4 -One leg stand – hold for 5 seconds x 10 with each leg, alternate legs, no hand support

-Tandem walk – 6 steps both directions x 3 sets

-Cross-over walk – 6 steps both directions x 3 sets

-Combined forward/backward step – 10 steps each side, one leg at a time, with support
