# Supplementary figures and images for: A feasibility randomized controlled trial of a community-level physical activity strategy for older adults with motoric cognitive risk syndrome
Source: Front Aging. 2024 Aug 8;5:1329177. doi: 10.3389/fragi.2024.1329177 (PMC11339030; doi:10.3389/fragi.2024.1329177)

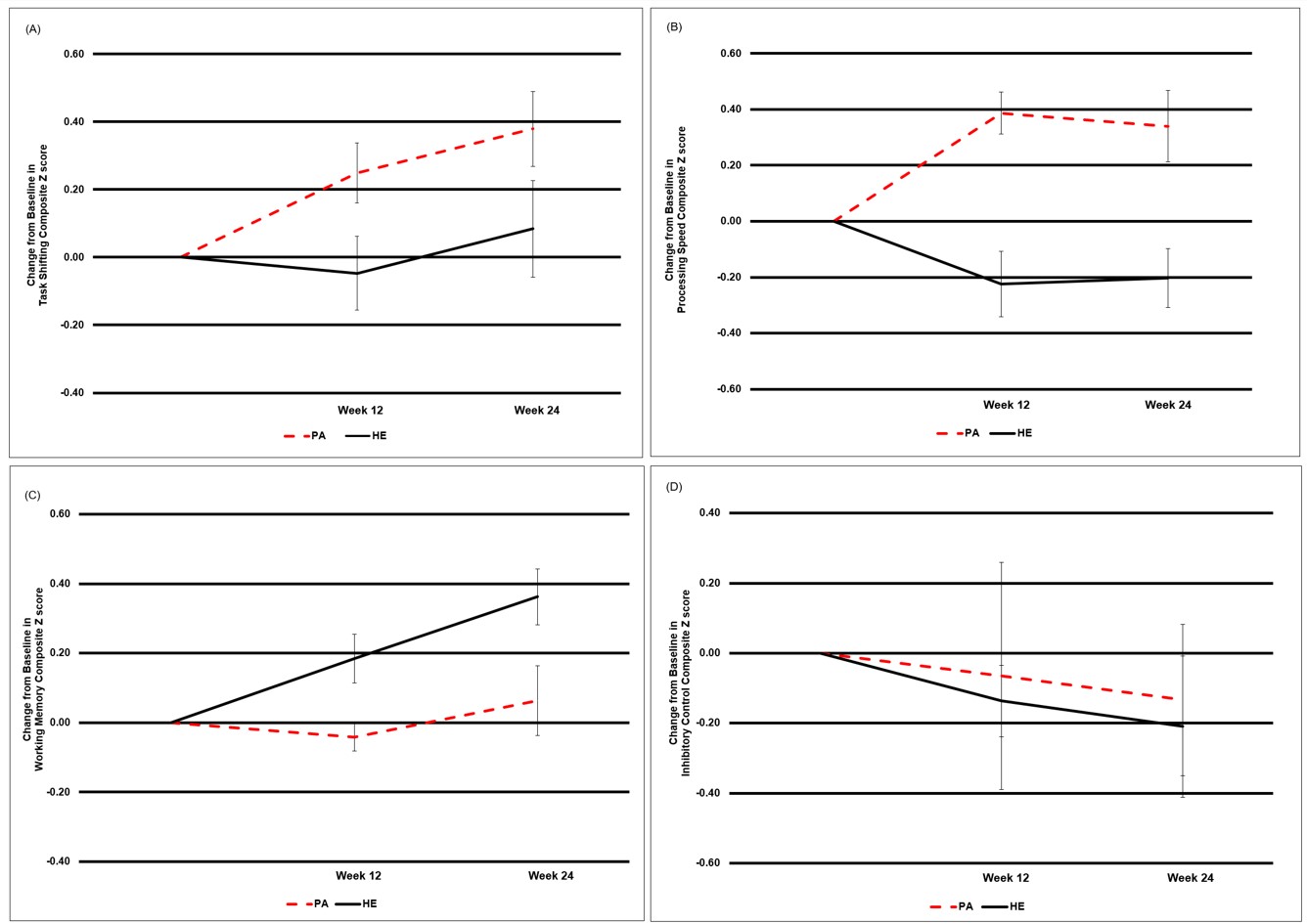

Supplement: Supplementary file 2 [file Image1.jpeg]
