# Supplementary material for: A feasibility randomized controlled trial of a community-level physical activity strategy for older adults with motoric cognitive risk syndrome
Source: Front Aging. 2024 Aug 8;5:1329177. doi: 10.3389/fragi.2024.1329177 (PMC11339030; doi:10.3389/fragi.2024.1329177)
Supplement: Supplementary file 3 [file DataSheet1.pdf]

ENGAGE-B
Physical Activity Program

Training Overview

- Physical Activity Program Features
- Safety
- Presentation/Video
  - Walking
  - Lower extremity strengthening
  - Flexibility
  - Balance training
- Restarting after Medical Event

- Demonstration / Practice

Features of the Program

- Age-appropriate, evidence-based
- Ability to be individualized or administer in group setting
- Can be conducted anywhere: home, senior center, community-center, assisted living, etc.
- Focus is on Mobility and Walking!
- Not complex
- Minimal low cost equipment
- Social interaction
- Group: one trainer for up to 4-6 participants

Safety

- Before starting the physical activity program, review person's:
  - Relevant medical history
  - Current medications
  - Emergency contact information
  - Current physical activity experience

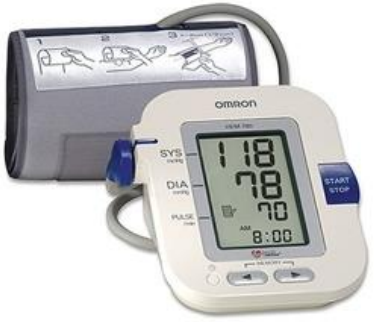

- At each session:
  - Start every session with a quick "check-in"
    - Recent medical events/change in medications
    - Look for significant, confusion, disorientation, problems with judgment or bizarre behavior
  - Vital signs before/after aerobic component, more if necessary
  - Stop if any contraindications observed
  - Monitor through out session using observations, RPE, changes in vitals

Environmental Safety

- Location: adequate space free of tripping/slipping hazards available, balance supports (chairs, wall, countertop)

- Equipment:
  - BP/HR monitoring machine
  - Medical emergency plan
  - CPR/AED certified personnel

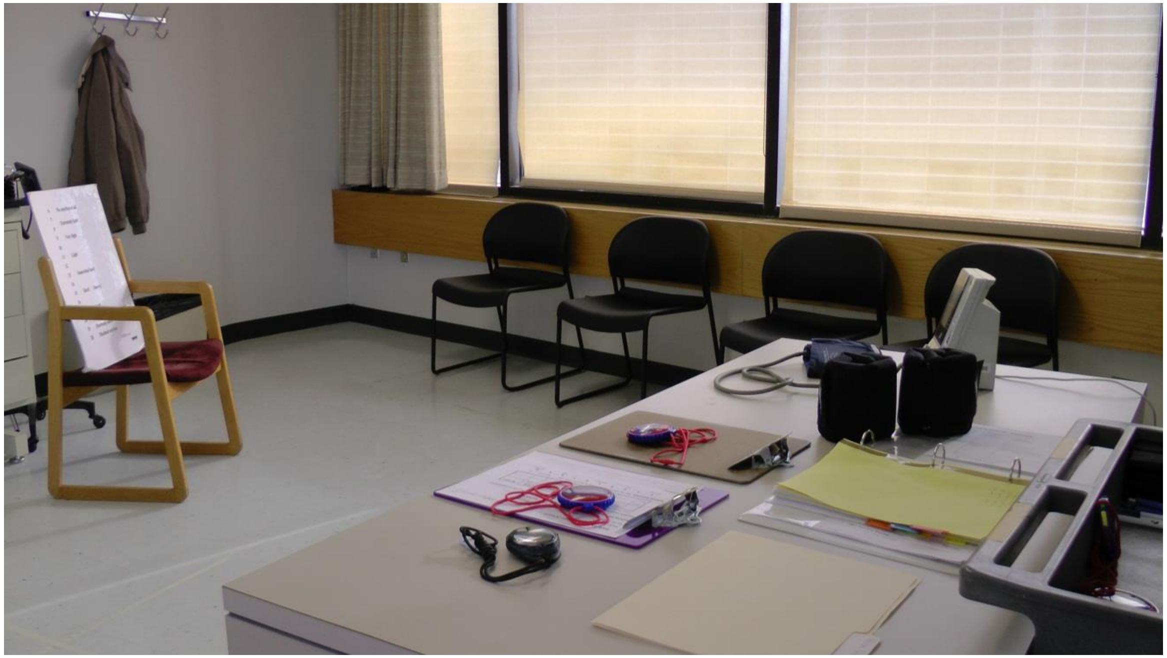

Contraindications to Exercise

- Resting blood pressure systolic > 200 mm Hg or diastolic > 100 mm Hg
- Decrease in systolic blood pressure ≥20 mm Hg following the activity
- Increase in systolic blood pressure to ≥250 mm Hg or in diastolic blood pressure ≥115 mm Hg following the activity
- Resting heart rate >120 bts./min or < 45 bts./min
- Increase in heart rate ≥90% of age predicted maximum
- Unusual or severe shortness of breath
- Chest pain including chest discomfort or pressure, left arm pain, report of indigestion, or stomach discomfort
- Palpitations
- Light headedness, dizziness or feeling about to faint
- A previous physical activity session had to be discontinued because of other symptoms excluding musculoskeletal symptoms (e.g., knees, ankles, hips) reported by the participant.

Physical Activity Program

- Combination of aerobic and strength training – approximately 45-60 minutes
- Includes light stretching and balance exercises
- Equipment required: sturdy chair, stop watch, ankle weights

Intensity

- Assess using Borg RPE scale (6-20)
  - self “Reported Perceived Exertion”
- Aerobic Component:
  - Overall
  - Range 11-15
  - Goal RPE 13 -“somewhat hard”
- Strength Component:
  - Localized
  - Range 14-16
  - Goal RPE 15 – “hard”

rating	description
6	NO EXERTION AT ALL
7	
	EXTREMELY LIGHT
8	
9	VERY LIGHT
10	
11	LIGHT
12	
13	SOMEWHAT HARD
14	
15	HARD (HEAVY)
16	
17	VERY HARD
18	
19	EXTREMELY HARD
20	MAXIMAL EXERTION

For more information see [illegible]

Progression

- Goal is to accumulate up to 150 minutes of aerobic activity per week.
  - 2 x week sessions at senior center
  - Progress gradually; determine realistic goals as 150 minutes per week of walking may be unrealistic for many participants
  - Accommodate varying levels of fitness by altering rate of progression
- Start with low weight (RPE 10-12) until correct form is established
  - Typically, men – 3 lbs / women – 2 lbs

Recording of Training Data

- Walking minutes
- RPE values
- BP/HR

If applicable: distance walked, steps, calories

Example:

Daily Exercise Ratings (VIVE2) Name: __________ Date: __________ Week: __________

Pre BP: __________ Mid BP: __________ **Post BP:** __________

Pre HR: __________ Mid HR: __________ **Post HR:** __________

Walking Minutes: __________ **Mid Walk RPE:** __________

Strength Exercises: Write your Rating of Perceived Exertion (RPE: 6-20) for each set in the corresponding box

Ankle Weight (lbs): __________

	Chair stand	Knee Extension		Leg Curl		Side Hip Raise		Toe Stand
		Right	Left	Right	Left	Right	Left	
Set 1								
Set 2								

Balance Exercises: Rate the difficulty of today's balance exercises (circle one) Bal ex tier: __________

1	2	3	4	5	6	7	8	9	10
Very Easy		Easy		Moderate			Hard		Very Hard

Walking Component

- Primary mode of physical activity.
- Designate duration for each participant according to progression (15-30 mins avg.).
- Track time
- Assess mid-walk RPE (target RPE = 13)
  - Warm-up/cool down phase

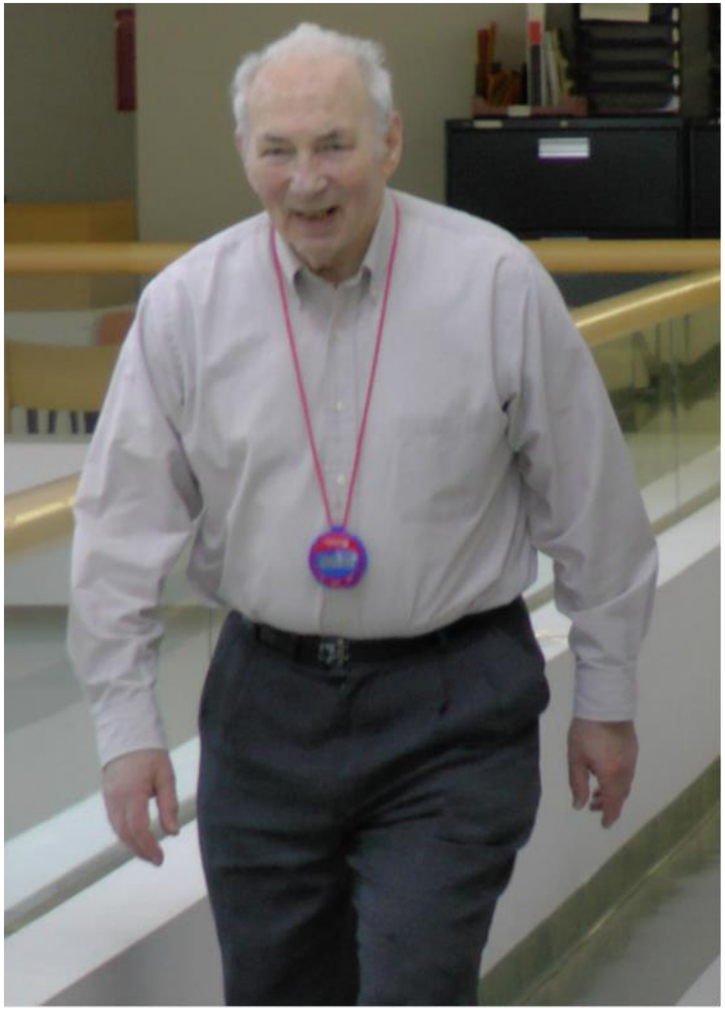

Video - Walk

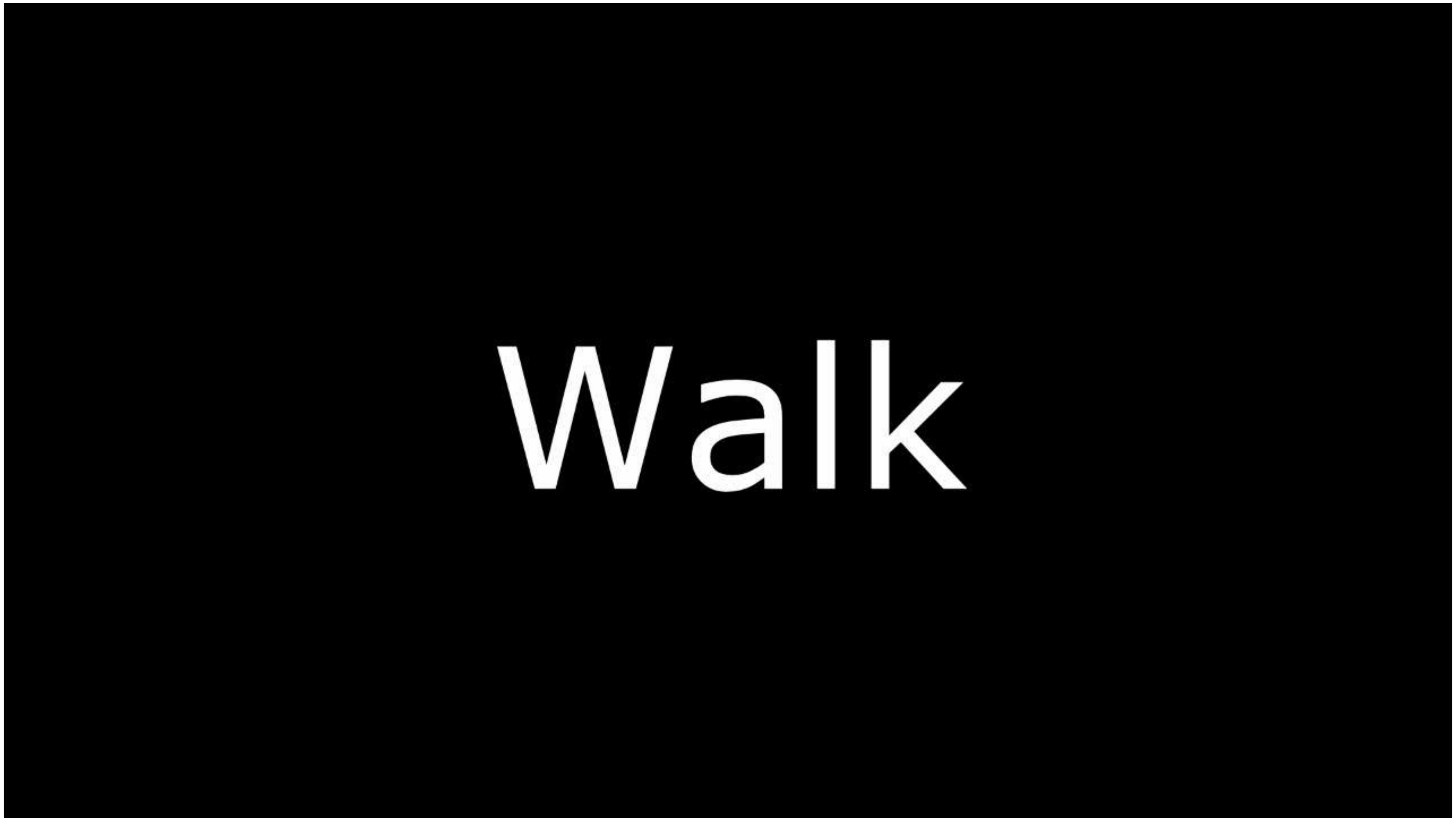

Stretches

- Static stretches (hold for 20-30 seconds).
- Four stretches: (1) hamstring and calf, (2) quadriceps, (3) chest and arms, (4)upper back.
- Complete directly after walking and before strength exercises.

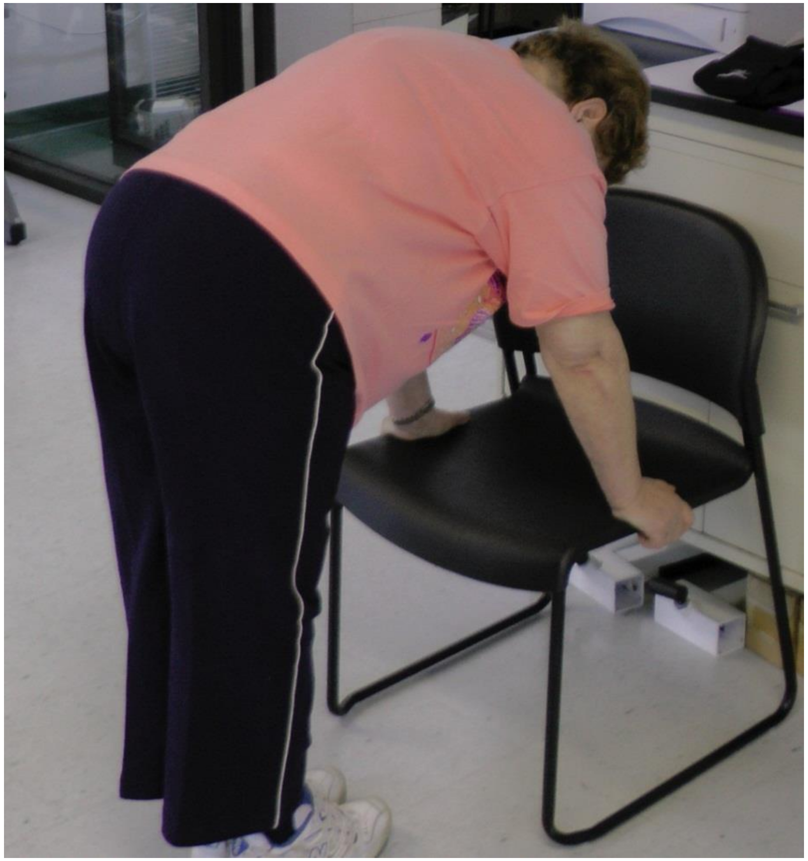

Strength Training

- Focuses on lower extremities.
- 2 sets x 10 repetitions with 60 sec rest between sets.
    1. Wide leg squat (chair stands)
    2. Knee extension (with ankle weights)
    3. Leg curls (with ankle weights)
    4. Side hip raise (with ankle weights)
    5. Toe stands
- Increase ankle weight in accordance with RPEs. Target RPE = 15.

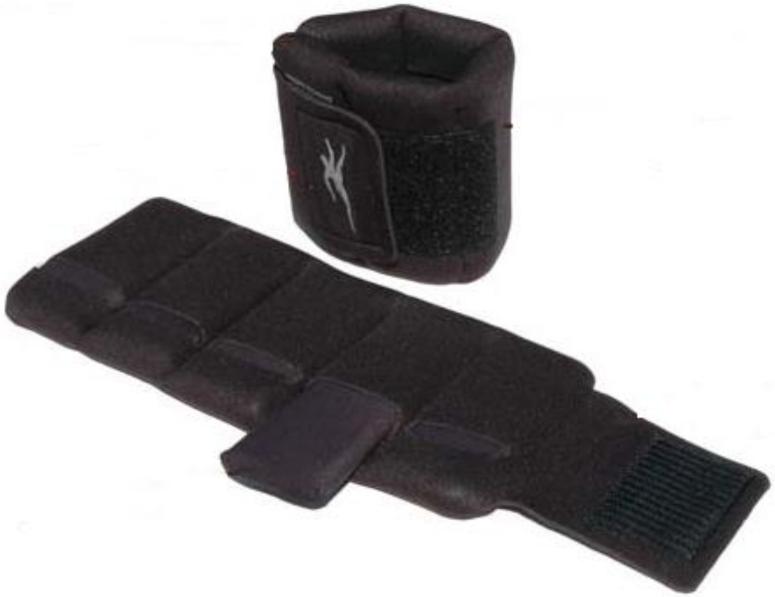

Balance Training

- 5 different tiers of increasing difficulty.
- Progress when performed correctly and without difficulty.
- About 5-8 minutes at end of class.

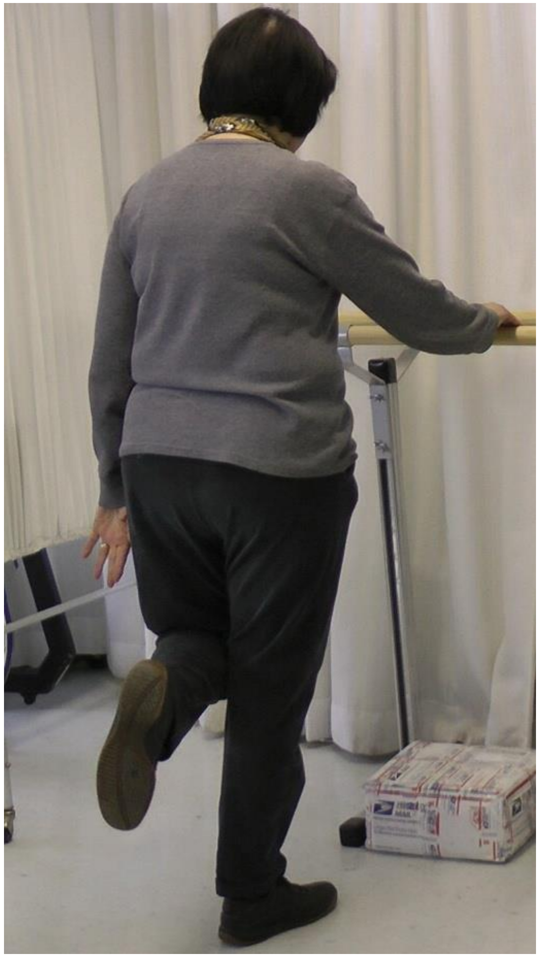

Stretches

- Hamstring and calf
- Quadriceps
- Chest and arms
- Upper back

Hamstring and Calf Stretch

- Bend forward at hip and rest hands on a sturdy chair.
- Legs straight, do not lock knees.
- Slight bend in elbows while keeping back flat.
- Hold 20-30 seconds.

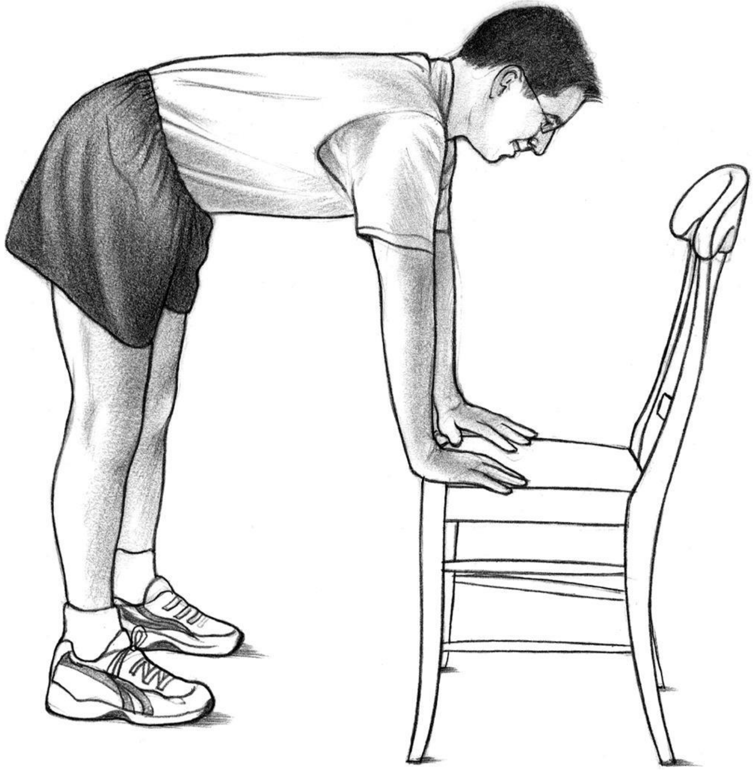

Quadriceps Stretch

- Stand next to sturdy chair or surface for balance.
- Bend right knee back and grab ankle or pant cuff with right hand. Hold on to chair with left hand for balance.
- If participant is unable to hold position, interventionist should hold leg as close to perpendicular as possible.
- Hold 20-30 seconds for both legs.

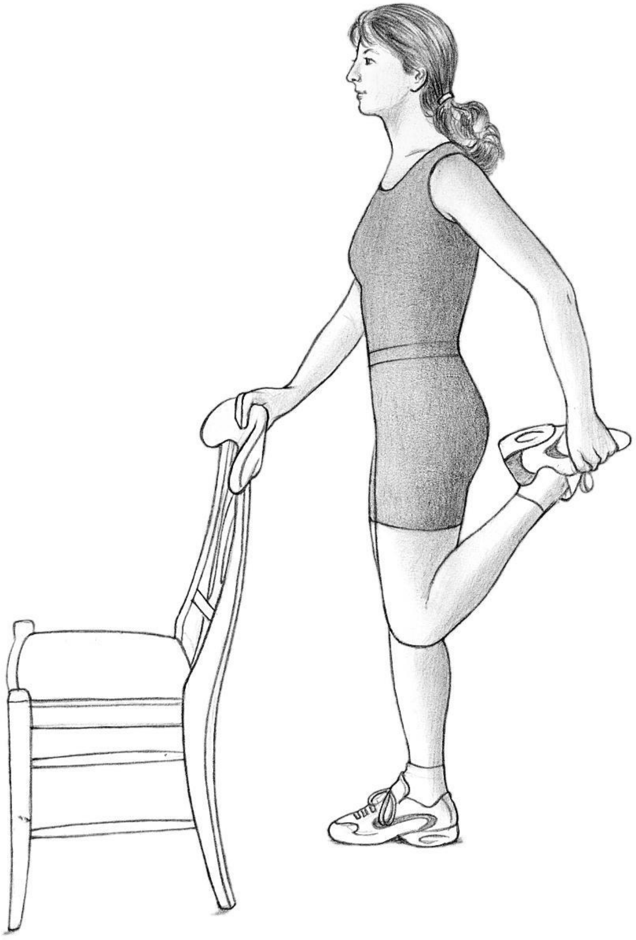

Chest and Arm Stretch

- Interlock fingers behind the back and extend arms.
- Keep arms straight, retract scapulae together, stick chest out.
- Hold 20-30 seconds.

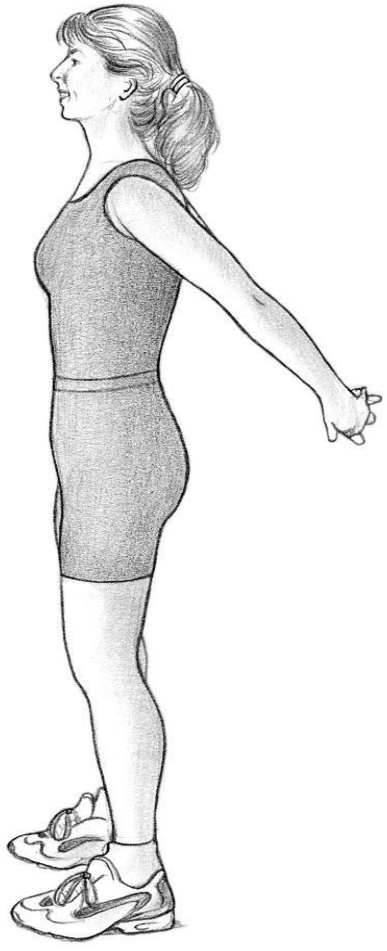

Upper Back Stretches

- Interlock fingers in front of the body and rotate palms away.
- Reach forward separating scapulae.
- Hold 20-30 seconds.

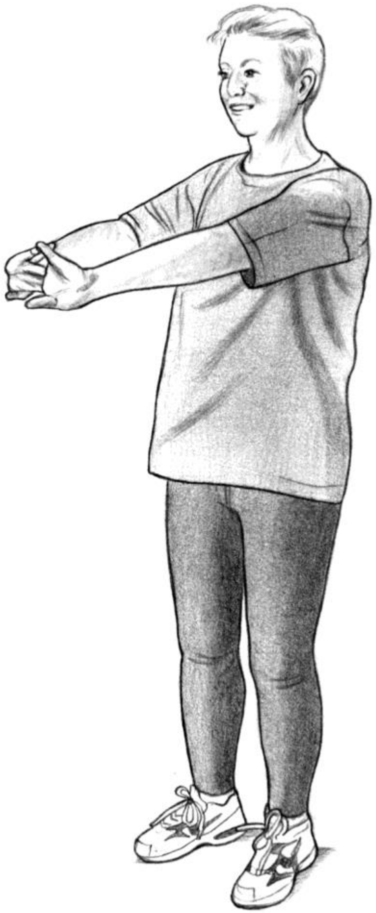

Strength Exercises

- Wide leg squat (chair stand)
- Knee extension
- Standing leg curl
- Side hip raise
- Toe stand

Wide Leg Squat

- Sit on chair with arms folded across chest and feet flat on floor.
- Stand up straight, exhaling on the way up, then sit back down completely.
- Keep chest up, knees should stay directly above ankle (do not move forward over toes)

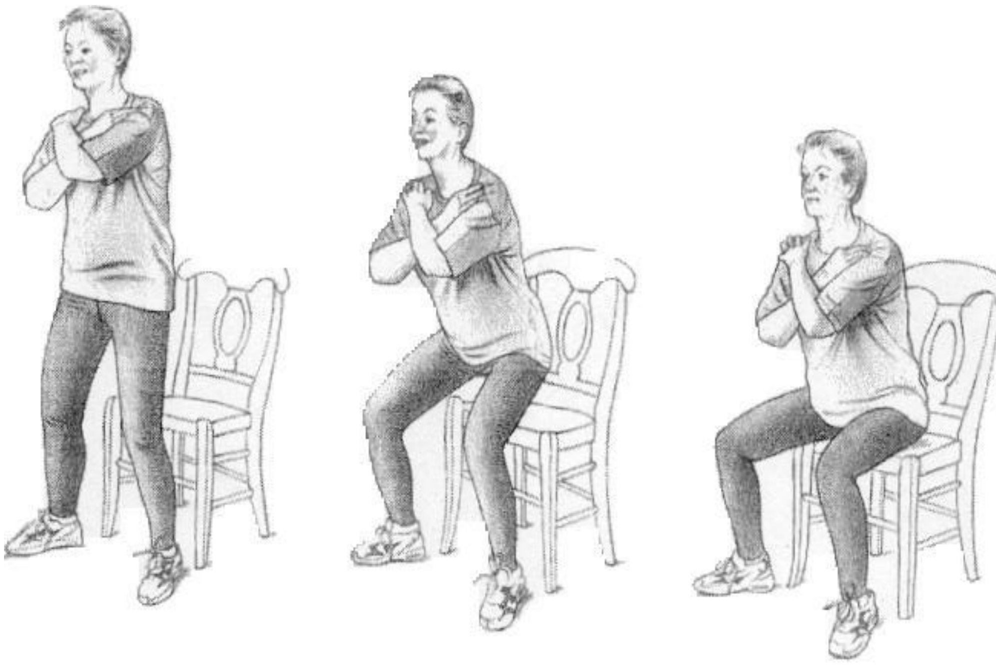

Video – wide leg squat

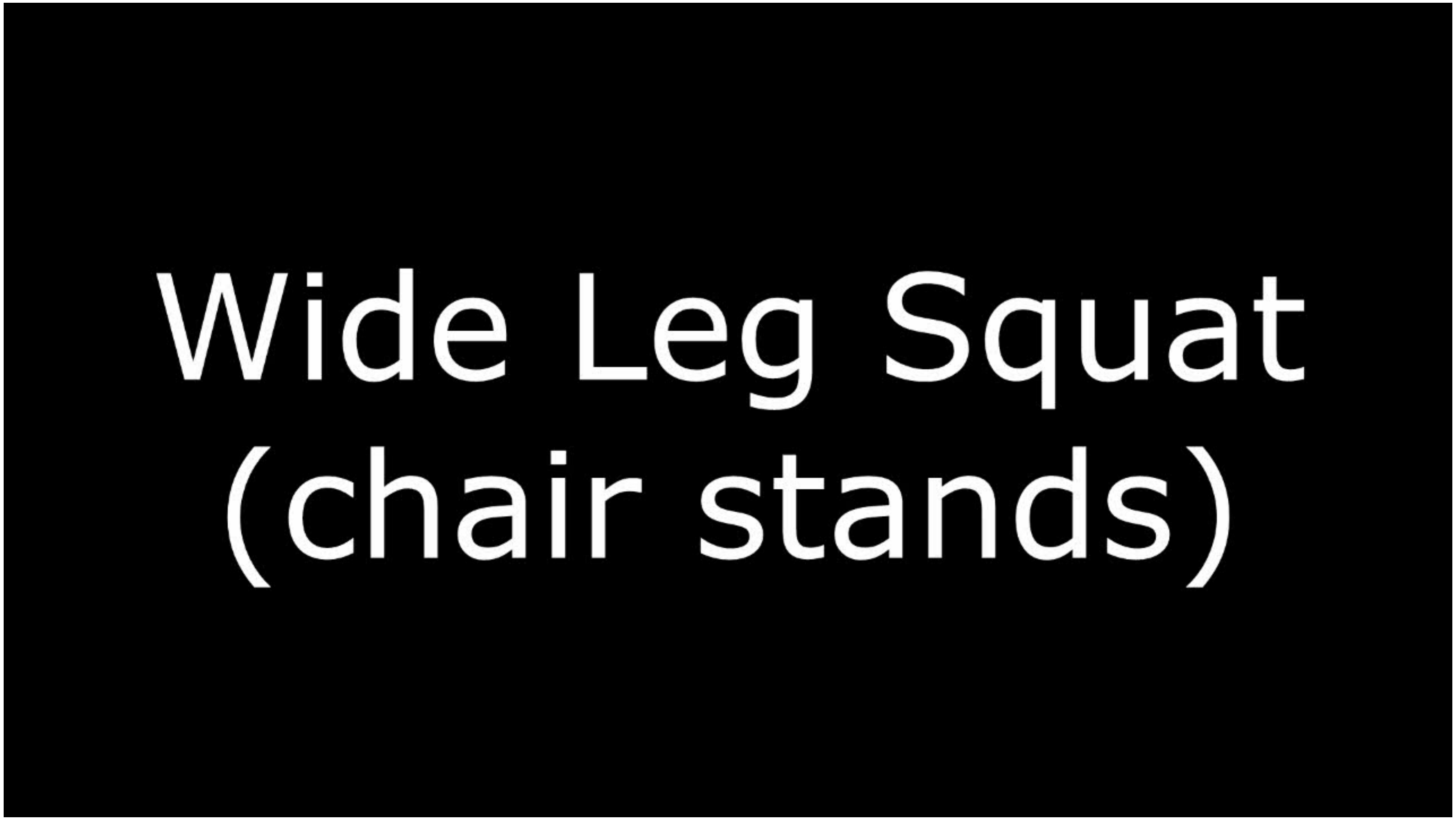

Knee Extension

- Seated with ankle weights.
- Place hands (or rolled up towel) under moving leg for improved range of motion.
- Extend leg so leg is as straight as possible then slowly lower back to ground.
- Complete 10 repetitions with right leg then switch to left leg.

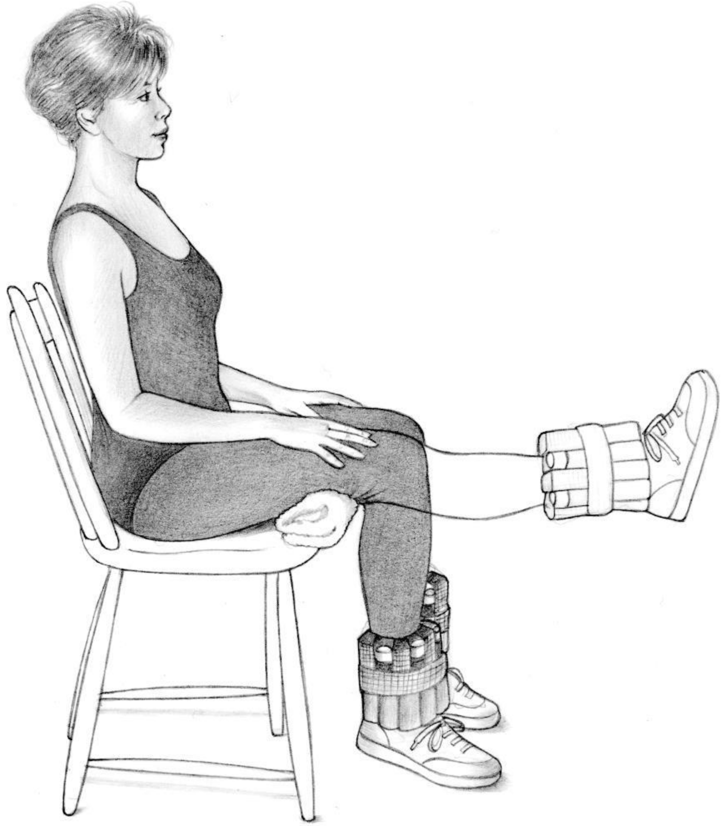

Video – Knee Extension

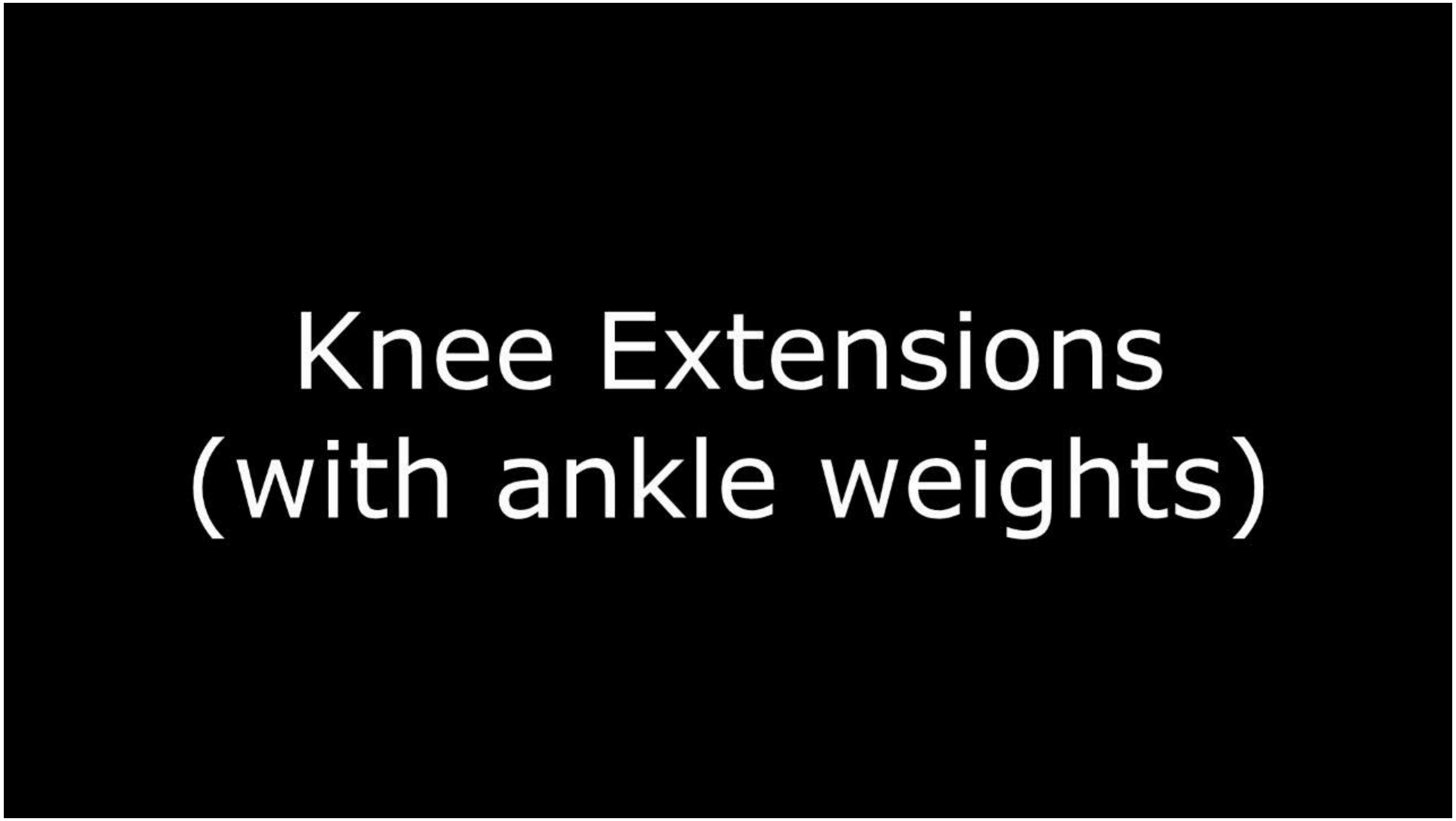

Standing Leg Curl

- With ankle weights, stand in front of a chair, wall, or ledge for support.
- Shift weight to left leg, lift right foot towards buttocks to ninety degrees then slowly lower to ground. Shift weight to right leg and repeat move with left leg.
- Alternate legs for 10 repetitions with each leg.

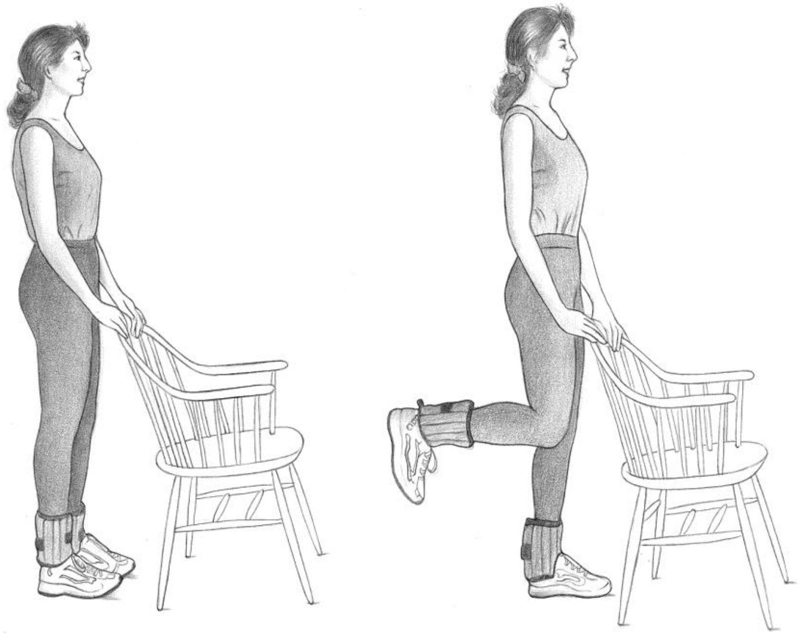

Video – Standing Leg Curl

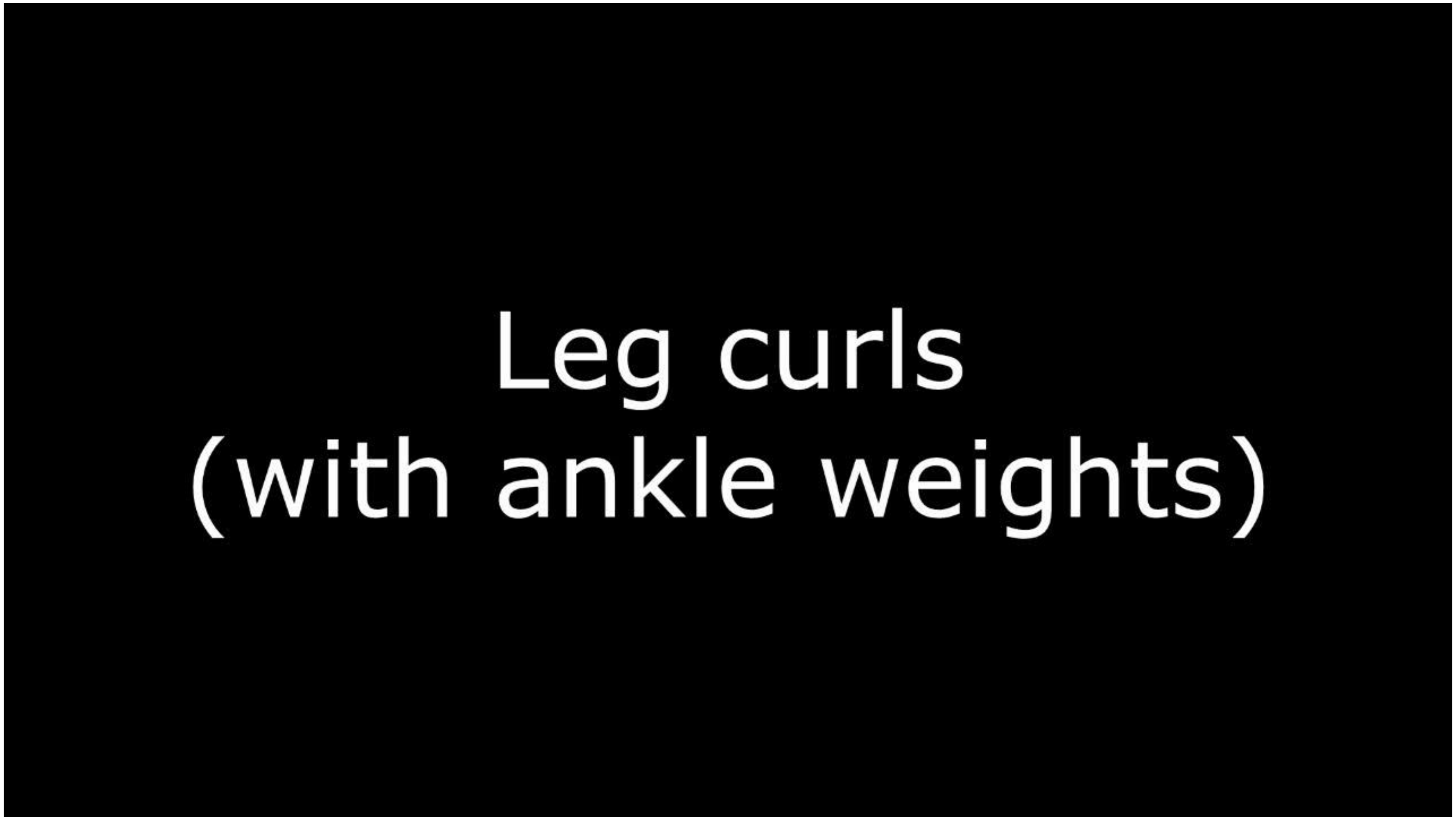

Side Hip Raise

- With ankle weights, stand in front of a chair, wall, or ledge for support.
- Abduct right leg until foot is 5-8 inches off the ground, pause at the top, then lower foot back to ground.
- Keep toes pointed straight ahead and stand upright (no leaning).
- Complete 10 repetitions with right leg then 10 with the left leg.

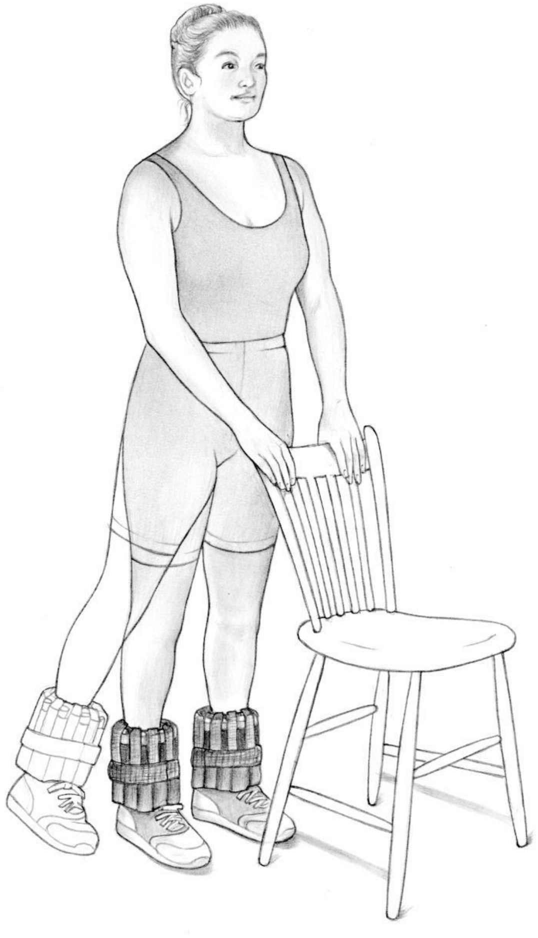

Video – Side Hip Raise

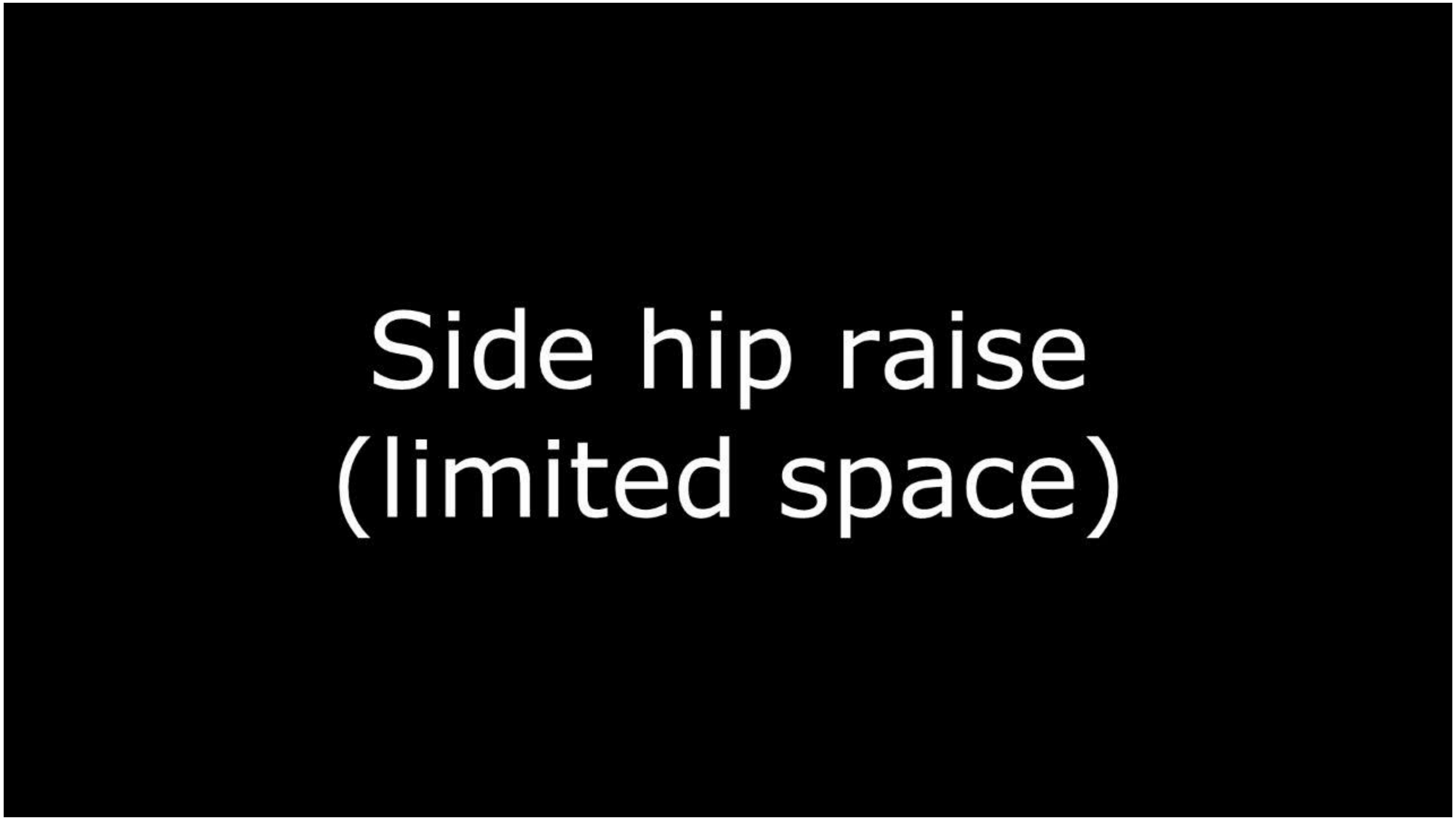

Toe stand

- Stand in front of a chair or wall for support.
- Raise heels off the ground as high as possible shifting weight to balls of feet. Slowly lower heels back to ground.
- Maintain good posture.
- To increase difficulty, do not hold on to chair (or wall).
- Do not use ankle weights.

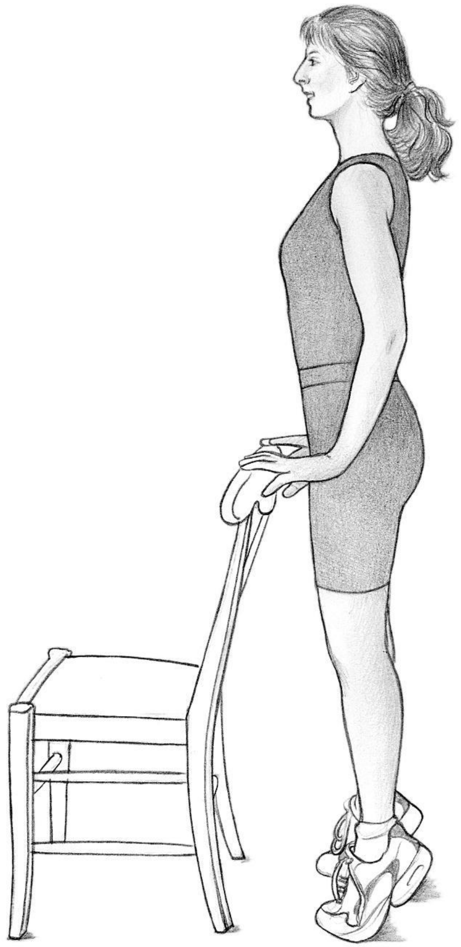

Toe Stands

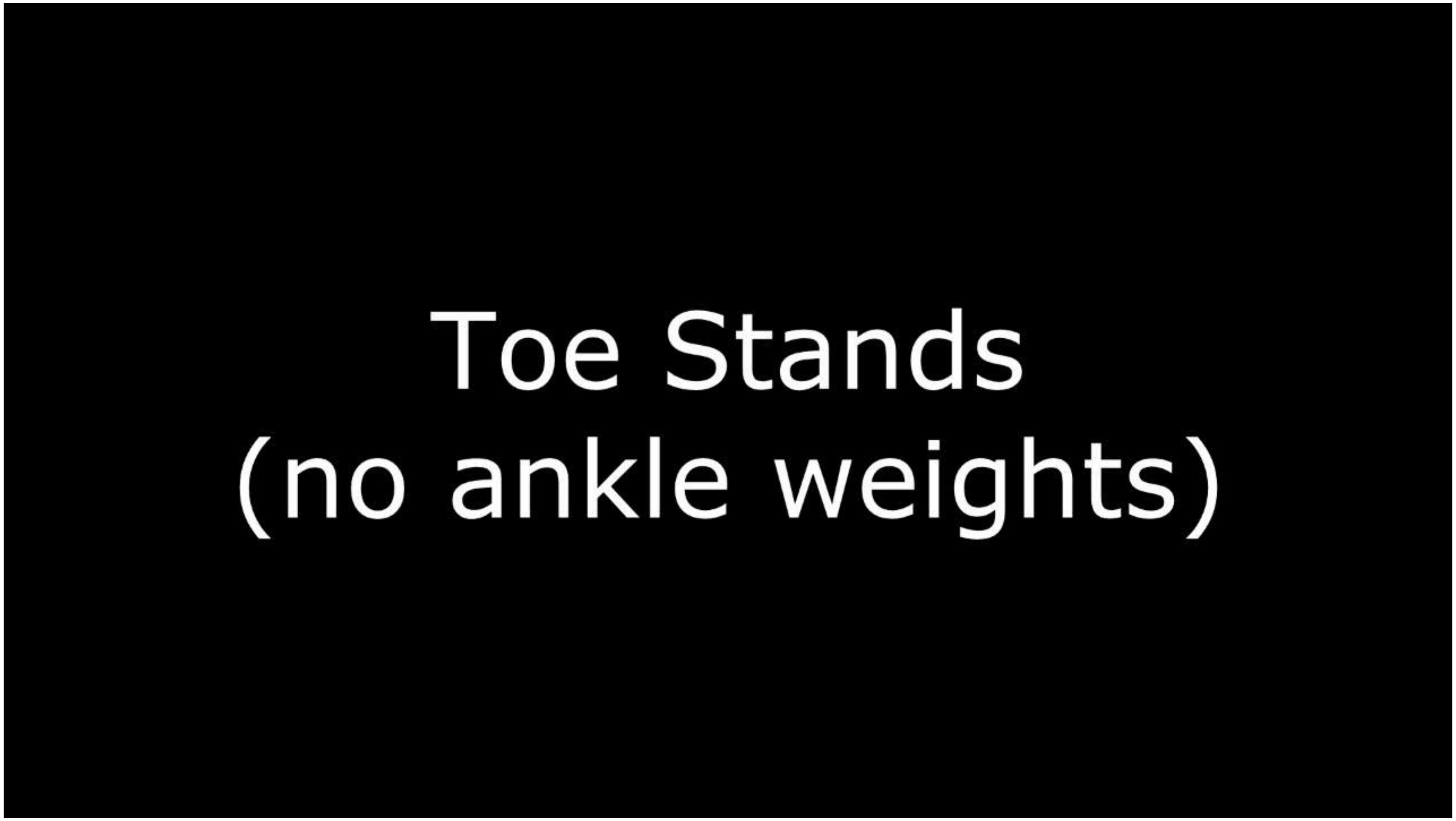

Balance Exercises

Balance Exercises

- Tier 1:
  - Hip circles – 5 each direction
  - Toe stands – hold 5 seconds x 10
  - Leg curls – hold 5 seconds x 10
  - Side step – 5 steps each direction, 5 times

Video – One Leg balance

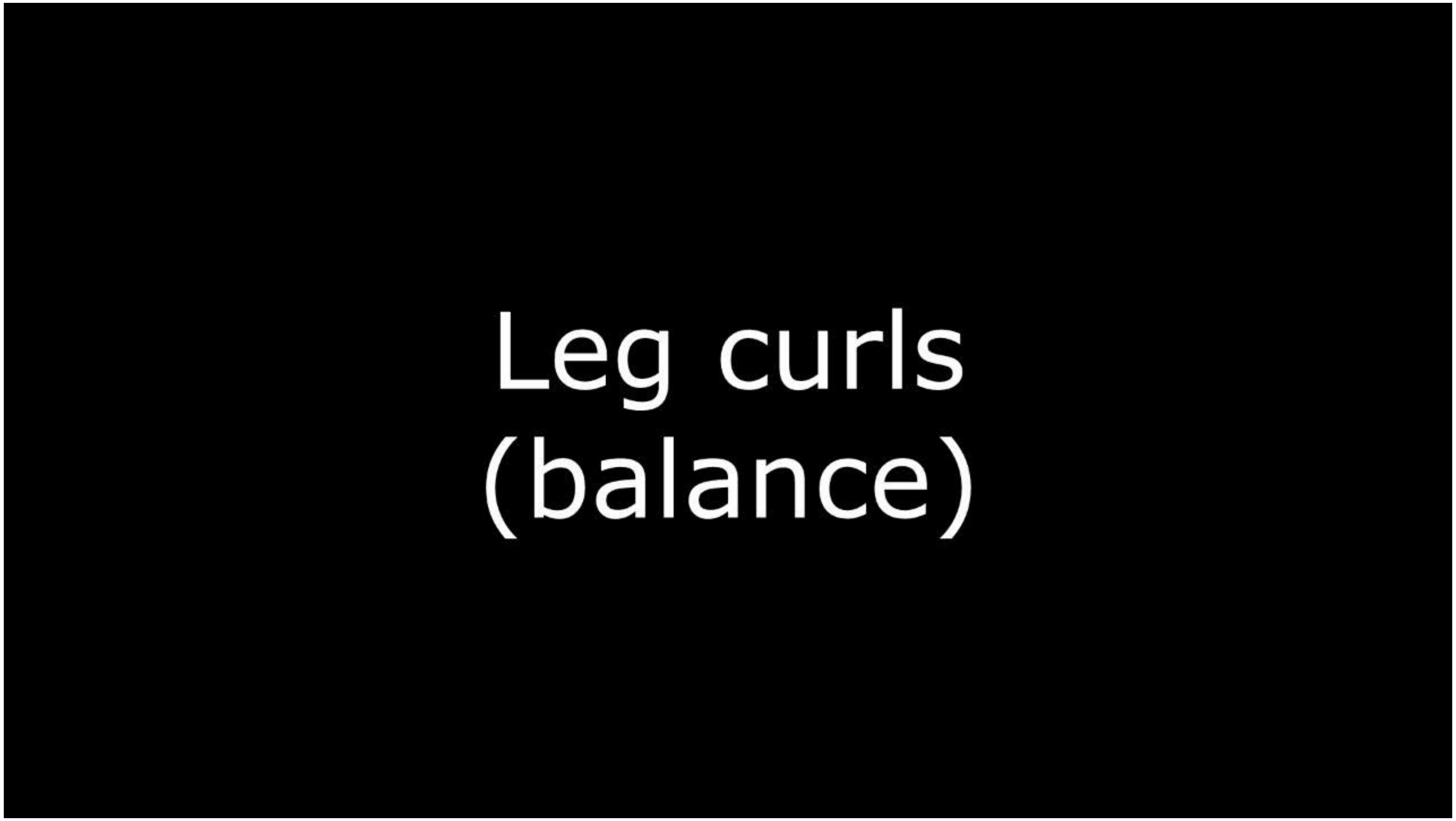

Balance Exercises

- Tier 2:
  - Toe stands (one hand)
  - Leg curls (one hand)
  - Forward steps – 10 each leg
  - Backward steps – 10 each leg
  - Side steps (one hand)

Video – Forward/Backward Steps

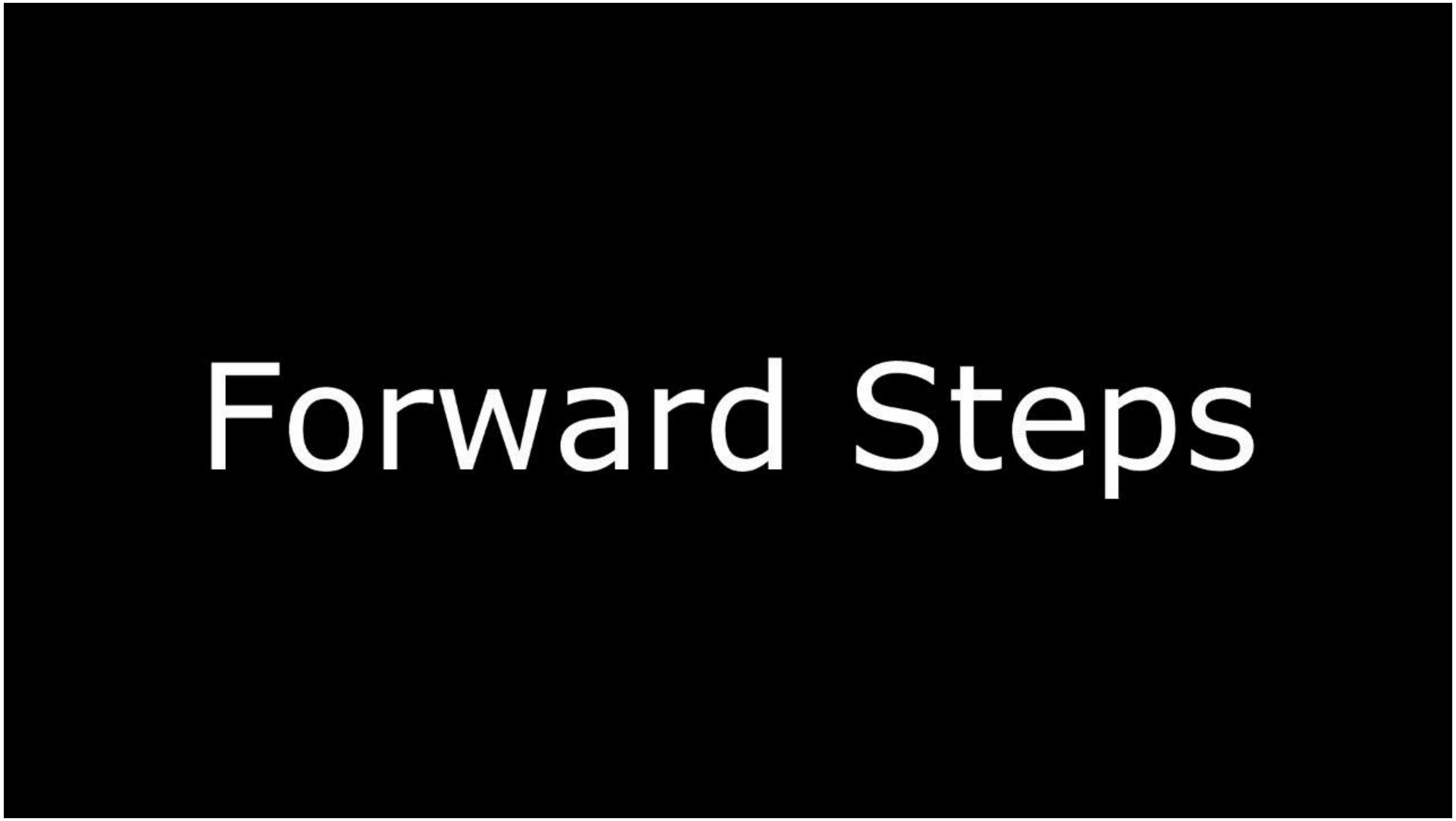

Balance Exercises

- Tier 3:
  - Crossover step – 5 each direction, 3 times
  - Side step (no hands)
  - Toe stands (no hands)
  - Forward steps (fingertips only)
  - Backward steps (fingertips only)

Video – Side Steps

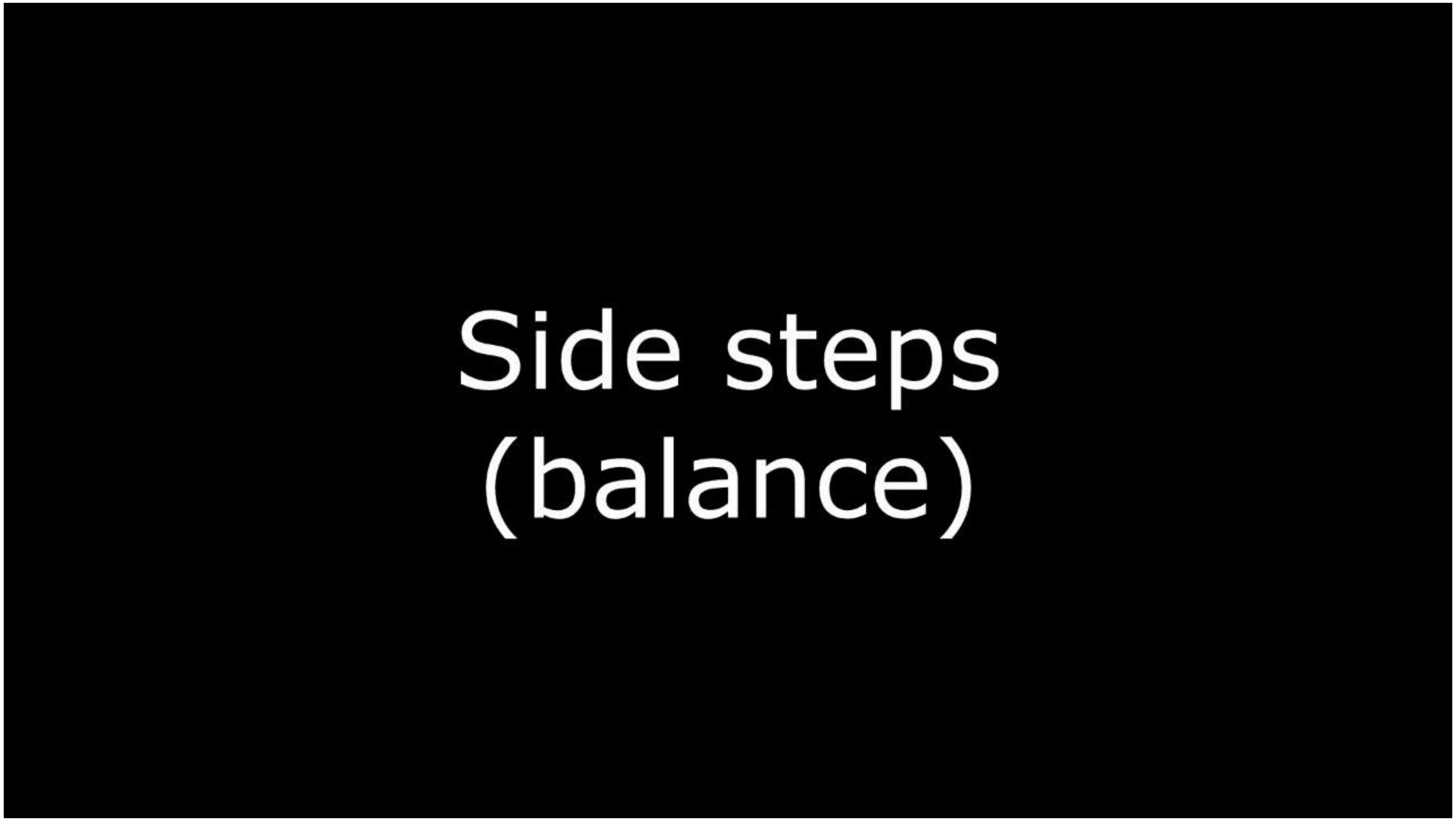

Balance Exercises

- Tier 4:
  - Leg curl (no hands)
  - Tandem walking – 6 steps each direction, 3 times
  - Cross-over walk – 6 steps each direction, 3 times
  - Step backward and forward – 10 times each leg

Resuming Physical Activity Following Medical Leave/Event

- Extended gaps in participation may occur
- Confirmation/documentation of approval from a medical professional
- Guidelines for restart of Physical activity:
  - Participant is able to leave home and walk independently a minimum of 4 meters (no more than straight cane)
  - Participant is not under prescribed activity, weight bearing limitations or physical rehabilitation
  - If physical activity is limited due to chest pain or dyspnea, restart will not occur without definitive treatment from the participant’s primary care provider
- Decision to return should be a collaborative process

Questions?

Demonstration / Practice
