# Supplementary material for: A feasibility randomized controlled trial of a community-level physical activity strategy for older adults with motoric cognitive risk syndrome
Source: Front Aging. 2024 Aug 8;5:1329177. doi: 10.3389/fragi.2024.1329177 (PMC11339030; doi:10.3389/fragi.2024.1329177)
Supplement: Supplementary file 4 [file DataSheet2.docx]

**Physical Activity intervention**

**Components of training**

The physical activity program will include aerobic, strength, flexibility, and balance training. We will focus on **walking** as the primary mode of physical activity. Other forms of endurance activity (e.g., stationary cycling, exercise video, etc.) are utilized when regular walking is contraindicated medically or behaviorally. Each session is preceded by a brief warm-up and followed by a brief cool-down period. In light of current clinical guidelines, participants are instructed to complete **flexibility** exercises following each bout of walking. Following a bout of walking, participants are instructed to complete a routine that focuses primarily on **lower extremity muscle strengthening.** **Balance training** is performed by all participants. In addition, the intervention will involve encouraging participants to increase all forms of physical activity throughout the day. This may include activities such as leisure sports, gardening, use of stairs as opposed to escalators, and leisurely walks with friends.

**Intensity of training.** The participants will be introduced to the intervention exercises in a structured way such that they begin with **lighter intensity and gradually increase** **intensity** over the first 2-3 weeks of the intervention. We will promote walking for physical activity at a **moderate intensity**. We will rely on **ratings of perceived exertion** and physical activity heart rate as a method to regulate physical activity intensity. Using Borg’s scale, that ranges from 6 to 20, participants are asked to walk at an intensity of 13 (activity perception SOMEWHAT HARD). They are discouraged from exercising at levels that approach or exceed 15 (HARD) or drop to a rating of 11 (FAIRLY LIGHT) or below. A set of lower extremity strengthening exercises are performed (2 sets of 10 repetitions) at an intensity of 15 to 16 using Borg’s scale for the strength training component of the program.

**Frequency and duration of training**

The intervention will consist of 2 x week sessions at the senior center with a general weekly walking goal of 150 minutes. This goal is **approached in a progressive manner.** We realize that for the current study population, 150 minutes per week may be an unrealistic goal, but participants should be encouraged to work up to this goal and walk at home if possible and also be advised that there are multiple ways that the goal can be achieved, based on the physical abilities and constraints of each participant.

**Aerobic component of the physical activity intervention**

**Selecting type of physical activity and intensities**

The primary physical activity is overground walking. Participants are taught to assess their Rating of Perceived Exertion Borg scale (RPE) using the Borg scale during the initial supervised physical activity sessions and periodically checked throughout to ensure continued understanding. We will promote a walking pace at a moderate intensity as defined as an RPE of 13. If the RPE drops to a rating below 11 or exceeds a rating of 15 the participant’s walking pace is adjusted. To ensure the goal of “moderate” physical activity intensity, physical activity heart rate will be monitored at weekly intervals during the study. Exercise training sessions will be terminated if the exercising heart rate is observed to be greater than 90% of age-predicted maximal heart rate. The participant will subsequently be reevaluated for medical contraindications to exercise and re-assessment of their exercise intensity goals. In addition, blood pressure and heart rate are monitored before and after physical activity.

The walking portion of the intervention should be performed in an area where the participant can be observed by the interventionist at all times, and there is access to a telephone and other emergency equipment.

**Strength training component of the physical activity intervention**

**Overview**

Strength training will focus primarily on five lower extremity exercises.

**Physical environment:** A sturdy chair and a medium bath towel are needed for each participant. The ideal chair should have a firm seat with no arms and the chair should be high enough so that when participants sit all the way back, their feet barely touch the floor. The back of the chair should be high enough so that participants can hold onto it while standing behind it. The towel can be placed under the knees during the seated knee extension exercises to raise the participant’s feet off the floor.

**Training Program**

The goal is to have participants complete the strength training at an intensity of 15 to 16 on the RPE scale.

**Specific strength training exercises**

The strength training intervention will consist of the following exercises:

Strength Exercise 1: Wide Leg Squat

Strength Exercise 2: Standing Leg Curl (with ankle weights)

Strength Exercise 3: Knee Extension (with ankle weights)

Strength Exercise 4 Side Hip Raise (with ankle weights)

Strength Exercise 5: Toe Stand

# Intensity and progression:

# For each strength exercise the subjects are instructed to perform 10 repetitions (1 set), rest for 1 minute and then perform a second set. For the leg curl, knee extension, and side hip raise exercise, the participants are instructed to perform each set of ten and then alternate legs. This will minimize the total time to perform the strength training exercises without compromising the quality of the program. The intensity and progression of the strength training program are monitored using the rating of perceived exertion (RPE) scale. Subjects are educated in the use of the RPE scale and report their individual RPE at the end of each exercise. For the strength training exercises, the participants should be instructed to report a “localized” RPE for the muscle groups involved in the particular exercise.

Selection of appropriate weight and progression: For each strength exercise that uses the ankle weights (leg curl, knee extension, side hip raises), the appropriate starting weight is determined by the study interventionist. At the start of a participant’s physical activity program they will be given a pair of ankle weights to use for training. Initially the ankle weights will contain a small amount of weight (3 lbs for men and 2 lbs for women). During the introduction to the strength training portion of the physical activity intervention, the interventionist will orient the subject to strength training and begin with weight settings that are “light” (RPE 10 to 11) and easy for the subject to accomplish. Participants should also be advised to wear comfortable clothing when performing their strength training exercises and that a comfortable pair of socks is advisable to prevent the development of skin irritation around the lower leg where the ankle weights are attached.

It is imperative for participants to complete the strength training at the proper intensity to maximize the training benefits. Intensity can be gauged using the RPE Scale (See forms). This scale ranges from 6 to 20 and is used to rate the difficulty of lifting a given weight. The participants should report a local RPE for the active muscle groups performing the exercise. The rating is determined for each exercise after completion of the second set of 10 repetitions. The training goal is an RPE of 15 to 16 (“HARD”). This intensity will be achieved in a progressive manner depending on the progress of the subject and the discretion of the exercise interventionist. Some subjects may require a longer period of accommodation to the strength training exercises.

Proper breathing techniques are essential for the safe and appropriate performance of the strength training exercises. Subjects should be instructed to avoid holding their breath and/or performing the “Valsalva maneuver” during training. Subjects are instructed to breathe through their mouths continuously and regularly throughout the exercises. This can be done in one of two ways. First, participants may count out loud to keep the pace of the exercises. Talking (counting) ensures that participants are not holding their breath. The second method entails inhaling before the lift, exhaling through the mouth while lifting, often referred to as “exhale during the exertion,” and inhaling through the nose during the lowering phase.

It is important for participants to start out at an easy level for all of these exercises. When the weight is light, the participant can safely learn the correct form of each strength exercise and learn how to breathe properly. After mastering proper technique, the participants can start to progress and meet the appropriate intensity for an effective workout.

# The strength training protocol

# STRENGTH EXERCISE 1: Wide Leg Squat

# *Starting position:*

The subject stands with their feet slightly greater than shoulder-width apart about 6-8 inches in front of a chair with their arms crossed in front of their chest with shoulders relaxed.

## The move:

1. Leaning slightly forward at the hip, the subject aims their buttocks into the chair and slowly lowers themselves back to a seated position. During this exercise, keep their chest up (lifted) and their back, neck, and head in a straight line.
2. Pause for a breath in the seated position.
3. Leaning slightly forward, they should stand up slowly, making sure to keep their knees directly above their ankles. As they do this, they should push up from their heels through their lower legs, thighs, hips, and buttocks, which will help keep their knees from moving in front of their feet.

# STRENGTH EXERCISE 1: Wide Leg Squat

## Notes for the study interventionist:

Subjects should be sure to keep their chests lifted throughout the move, so that the body doesn’t curl forward. Eyes should be looking straight ahead rather than down at the floor. If participants are experiencing any pain in their knees, interventionists should guide their technique to make sure they are not letting their knees move forward past their toes during the move and that the lower leg stays perpendicular to the floor. It is important to remind participants not to sit down in the chair completely. In addition, participants should be reminded to lower their bodies in a slow controlled manner during this exercise.

Make sure subjects:

- Lean just slightly forward when beginning the move
- Don’t allow their knees to come in front of their toes
- Tighten their abdominal muscles
- Don’t hold their breath

#
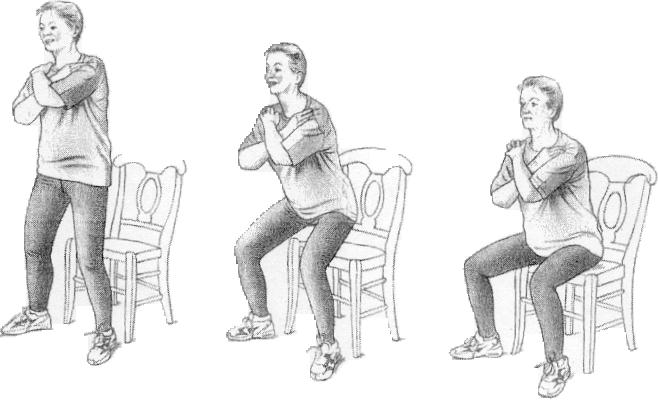


# STRENGTH EXERCISE 2: Standing Leg Curl (with ankle weights)

# *Starting position:*

Subject stands with their feet slightly apart behind a chair with their hands gently resting along the top of the chair back for balance. They are then instructed to shift their body weight to their left leg.

## The move:

1. Keeping their thighs side-by-side, slowly lift their right foot up towards their buttocks until their upper and lower leg form a ninety-degree angle.
2. Pause for a breath.
3. Slowly lower their right foot back to the ground, shift their weight to the right leg, and repeat the move with the left leg.

# STRENGTH EXERCISE 2: Standing Leg Curl (with ankle weights)

## Notes for the study interventionist:

Make sure the subjects:

- Keep thighs and hips even and knees touching
- Don’t arch their backs as they do the exercise
- Don’t let the knee or thigh move forward as the lower leg curls up
- Don’t hold their breath


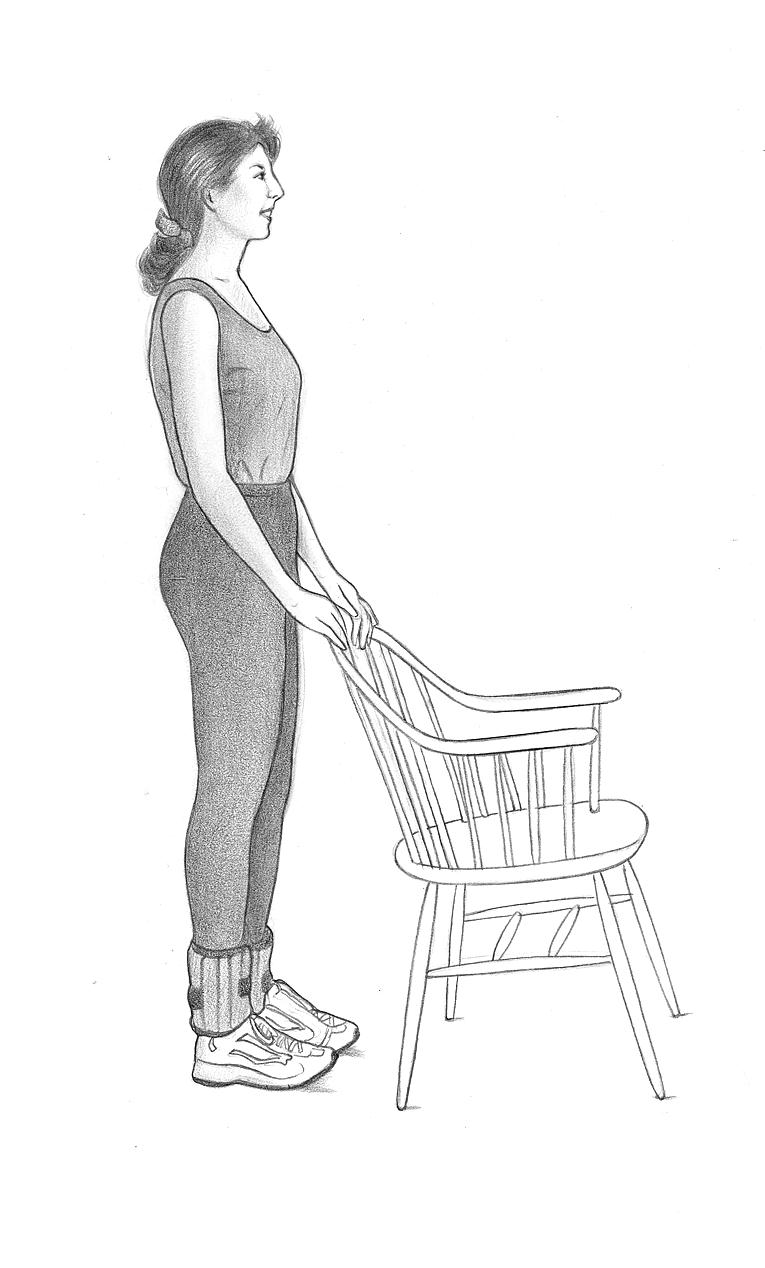


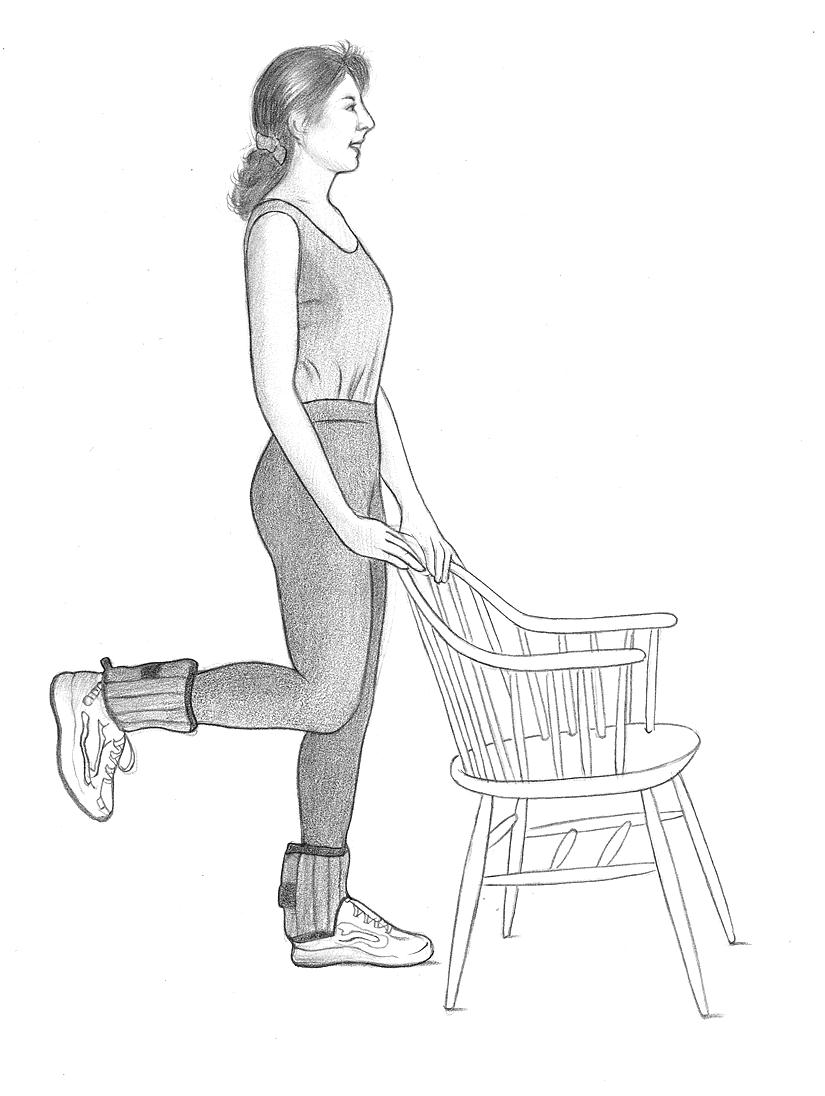


# STRENGTH EXERCISE 3: Knee Extension (with ankle weights)

## Starting position:

Subject is to sit back in a chair with their feet shoulder-width apart and knees slightly separated and directly above their feet. A rolled towel can be placed beneath the knees for comfort and to allow full range of motion during the exercise, as the toes should just brush against the floor when extending the leg. For participants who are extremely tall a set of stackable chairs can be used to raise the participant’s foot to just above the floor.

## The move:

1. Keeping their foot flexed, slowly raise their left leg until it is fully extended, with the knee as straight as possible.
2. Pause for a breath.
3. They are then instructed to slowly lower their left leg back to the ground.

##

# STRENGTH EXERCISE 3: Knee Extension (with ankle weights)

## Notes for the study interventionist:

Make sure participants:

- Don’t arch their backs
- Straighten their legs as far as possible at the end of the lift – the last part of the muscle contraction is the most important
- Don’t hold their breath


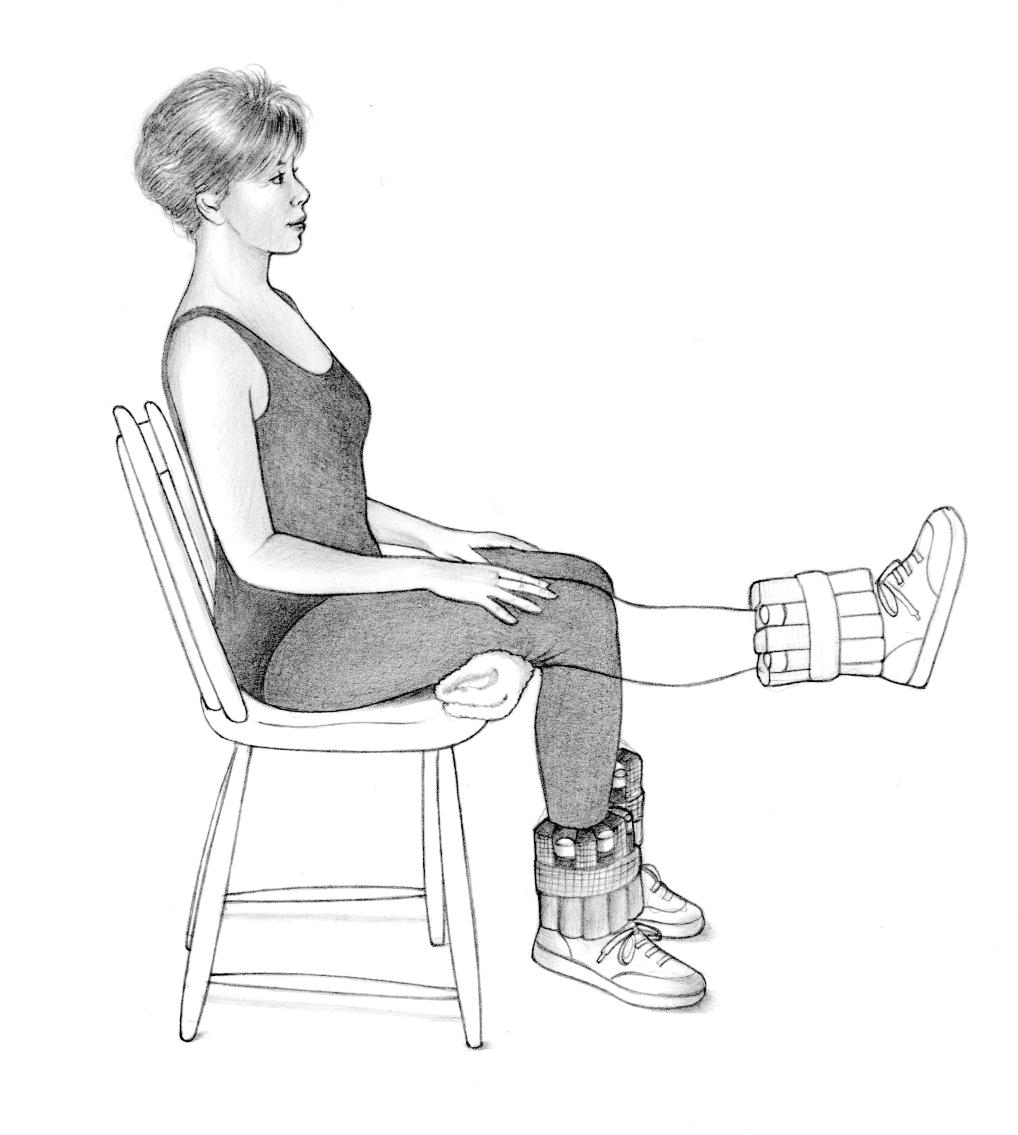


# STRENGTH EXERCISE 4: Side Hip Raise (with ankle weights)

## Starting position:

Stand straight with feet together and hands gently resting on the back of a chair for balance.

## The move:

1. Keeping their toes pointed straight ahead; slowly lift their right leg out to the side until their foot is 5-8 inches off the ground. Do not lock the knee on the supporting leg.
2. Pause for a breath.
3. In a comparison led motion, lower the right leg back to the ground.

## Reps and sets:

Complete 10 repetitions on both the right and left legs for the set, rest for about a minute, and then repeat for a second set of 10.

# STRENGTH EXERCISE 4: Side Hip Raise (with ankle weights)

## Notes for the program leader:

Make sure participants:

- Keep their torsos upright during this exercise, not leaning to one side
- Raise their legs no more than 12 inches off the ground
- Keep their fingertips on top of the chair for balance
- Don’t hold their breath

#


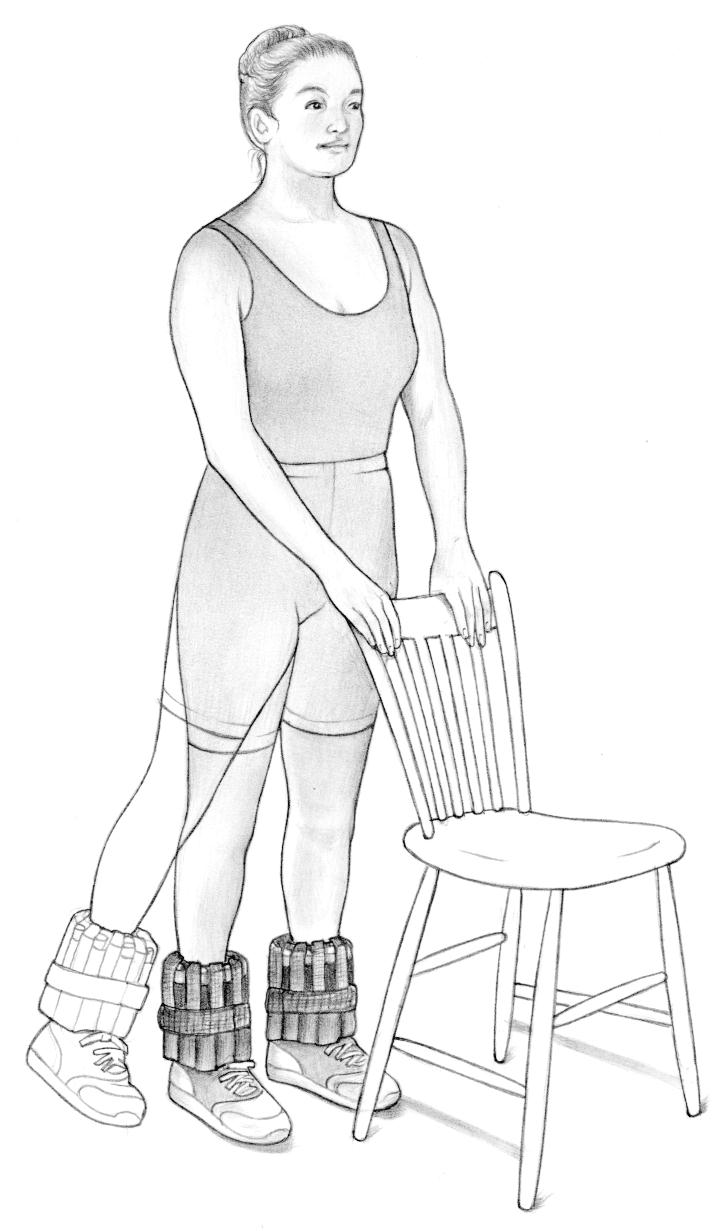


**STRENGTH EXERCISE** **5: Toe Stand**

The toe stand is an excellent physical activity that improves balance and ankle flexibility while also strengthening the gastrocnemius and soleus muscles in the back of the lower legs. As participants get stronger, they can progress from Level 1 to Level 2 of the move.

**Level 1:** Toe stand on both feet with hand support

**Level 2:** Same as Level 1 but without hand support

*Start with Level 1.*

If their calf muscles are weak or inflexible, they may not be able to raise their heels. They can improve the strength of these muscles until they can lift their heels fully. Then proceed to Level 2 – the same physical activity but without help from your hands.

## Starting position:

Stand 12 inches away from a wall (or back of a chair), with feet about 12 inches apart.

**Level 1:** Rest fingertips lightly on a wall (or back of a chair) to help maintain their balance.

**Level 2:** Same as Level 1 but without their fingertips actually touching the wall (or chair). For safety’s sake, always perform this physical activity with a wall (or chair) in front of you.

## The move:

1. Slowly raise them self as high as possible on the balls of their feet.
2. Hold the position for a slow count of three.
3. Slowly lower their heels back to the ground.

## Notes for the study interventionist:

Make sure participants:

- Maintain good upright posture
- Do the toe stands slowly—many people have a tendency to raise and lower themselves too quickly
- Don’t hold their breath

# STRENGTH EXERCISE 5: Toe Stand

#
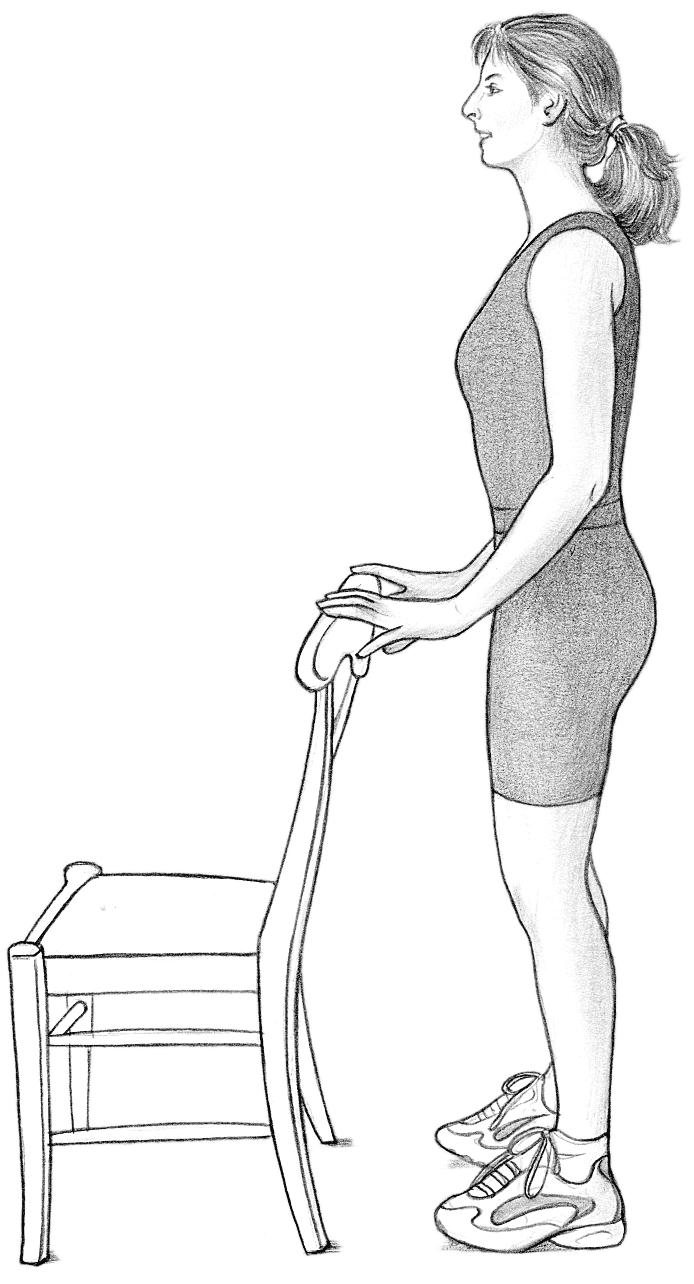

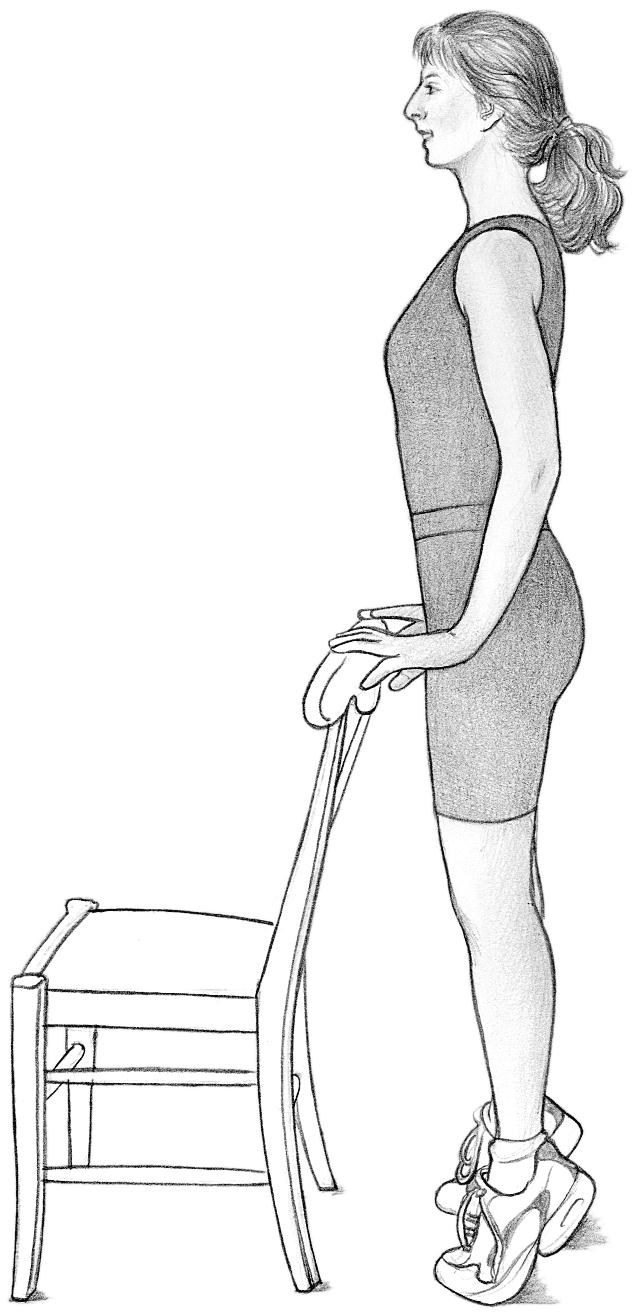
Cool-down

**Hamstring & Calf Stretch**

1. Stand facing a sturdy chair.
2. Slowly bend forward at the hip, keeping their legs straight without locking their knees. Rest your hands on the seat of the chair with their elbows slightly bent, feeling a stretch in the back of their upper and lower leg. Keep your back flat.
3. Hold the stretch for a count of 20-30 seconds.


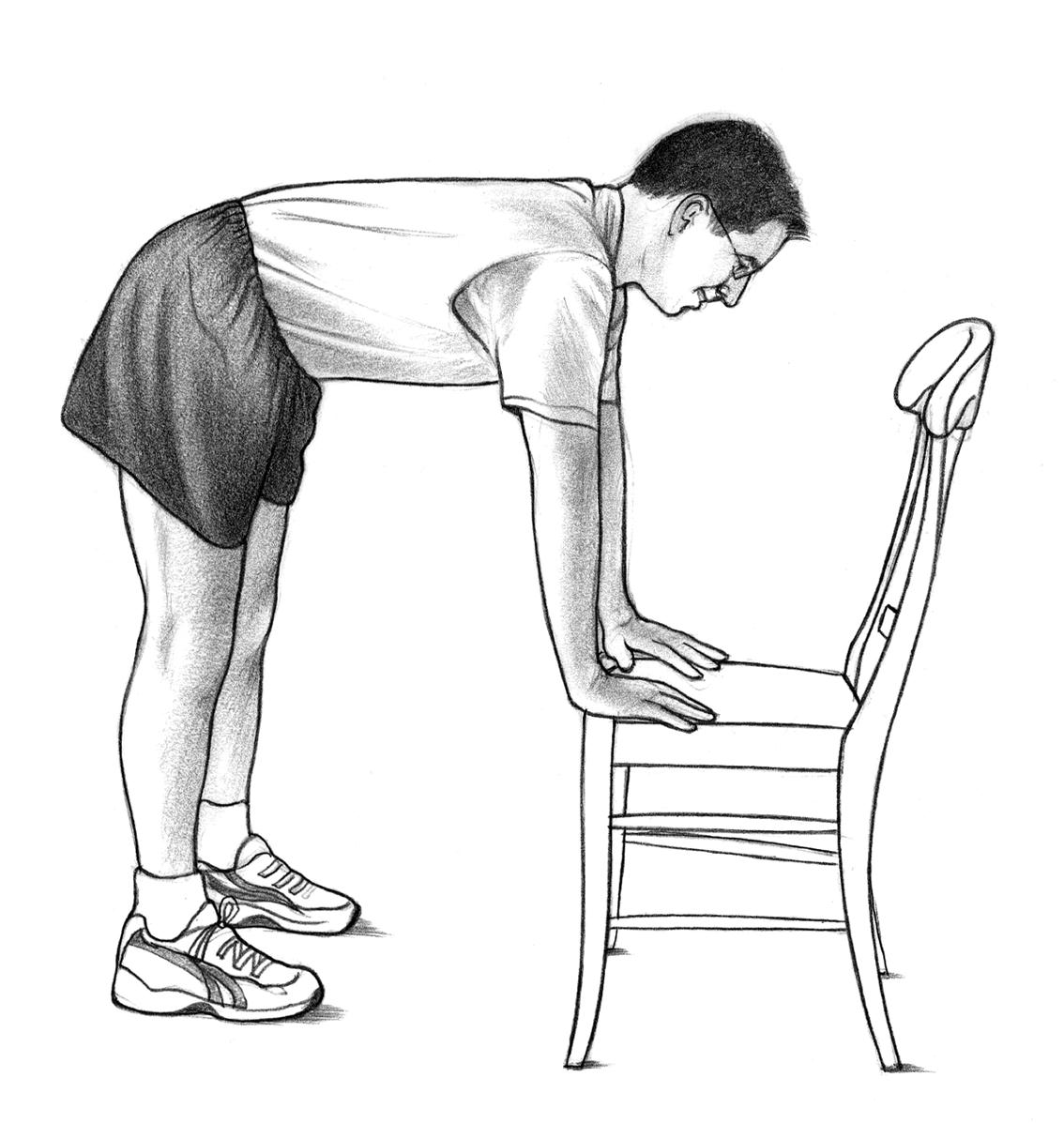


**Quadriceps Stretch**

1. Stand next to a sturdy chair with their feet about shoulder-width apart and their knees straight – but not locked.
2. Hold onto the chair for balance with their left hand. Bend their right leg back and grab their right foot or ankle in their right hand until their thigh is perpendicular to the ground. Make sure they stand up straight – don’t lean forward. (If they can’t grab their ankle in their hand, keep their leg as close to perpendicular as possible and hold the bend.) They should feel a stretch in the front of their thigh.
3. Hold the stretch for a count of 20-30 seconds, and then repeat the stretch with the other leg.


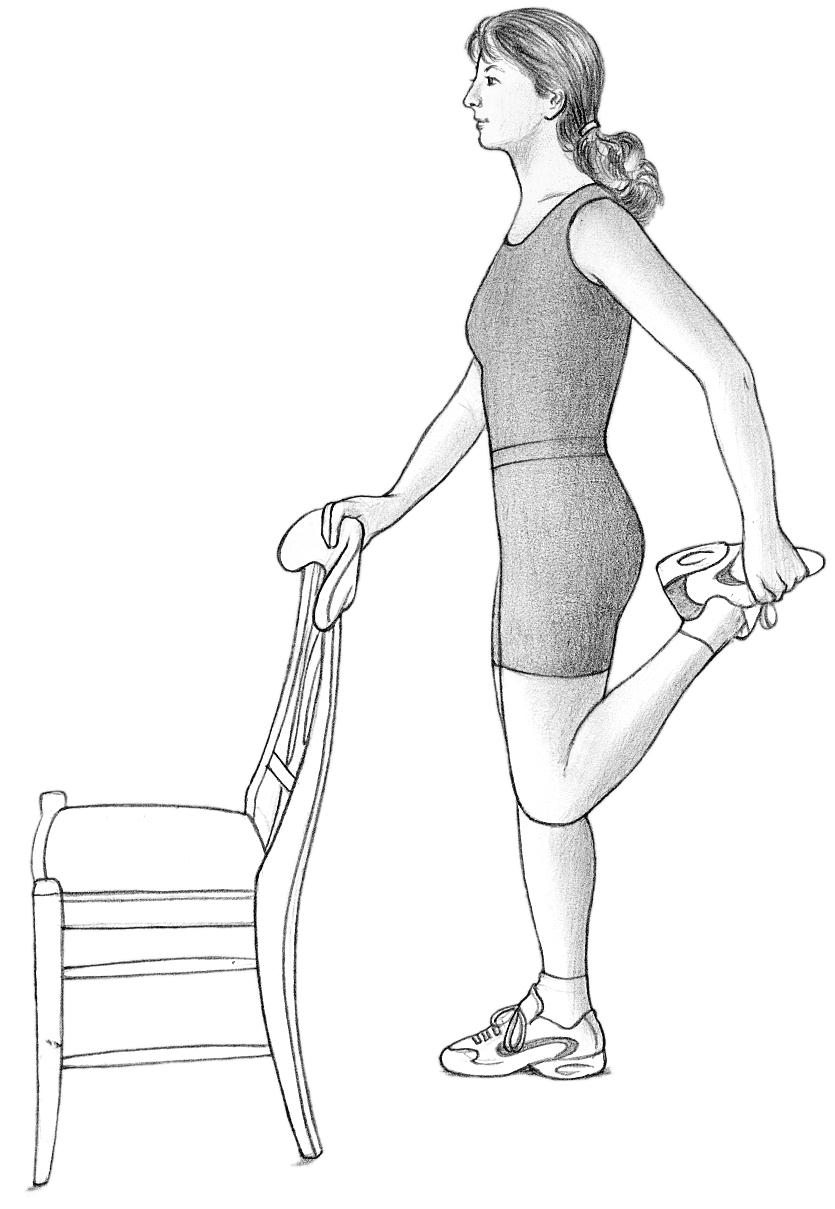


**Chest & Arm Stretch**

1. Stand with their arms down by your side.
2. Extend both arms behind you and clasp your hands together. Make sure their arms are straight before lifting them up behind you as high as possible. Keep their chest forward and shoulders back during the stretch.
3. Hold the stretch for a count of 20-30 seconds.


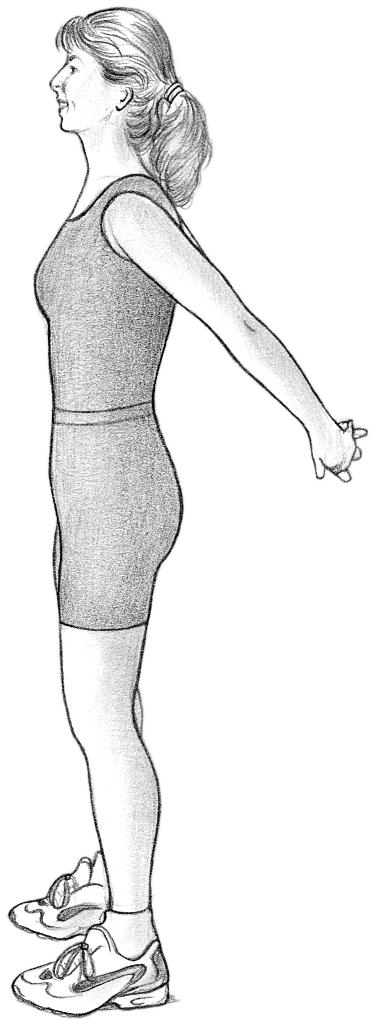


**Upper Back Stretch**

1. Stand (or sit) with their feet shoulder-width apart, their knees straight but not locked, and their hands clasped in front of themselves. Rotate their hands so that their palms face the ground. Then raise their arms to about chest height.
2. Press their palms away from their body and feel a stretch in your neck, upper back, and along their shoulders.
3. Hold the stretch for a count of 20-30 seconds.


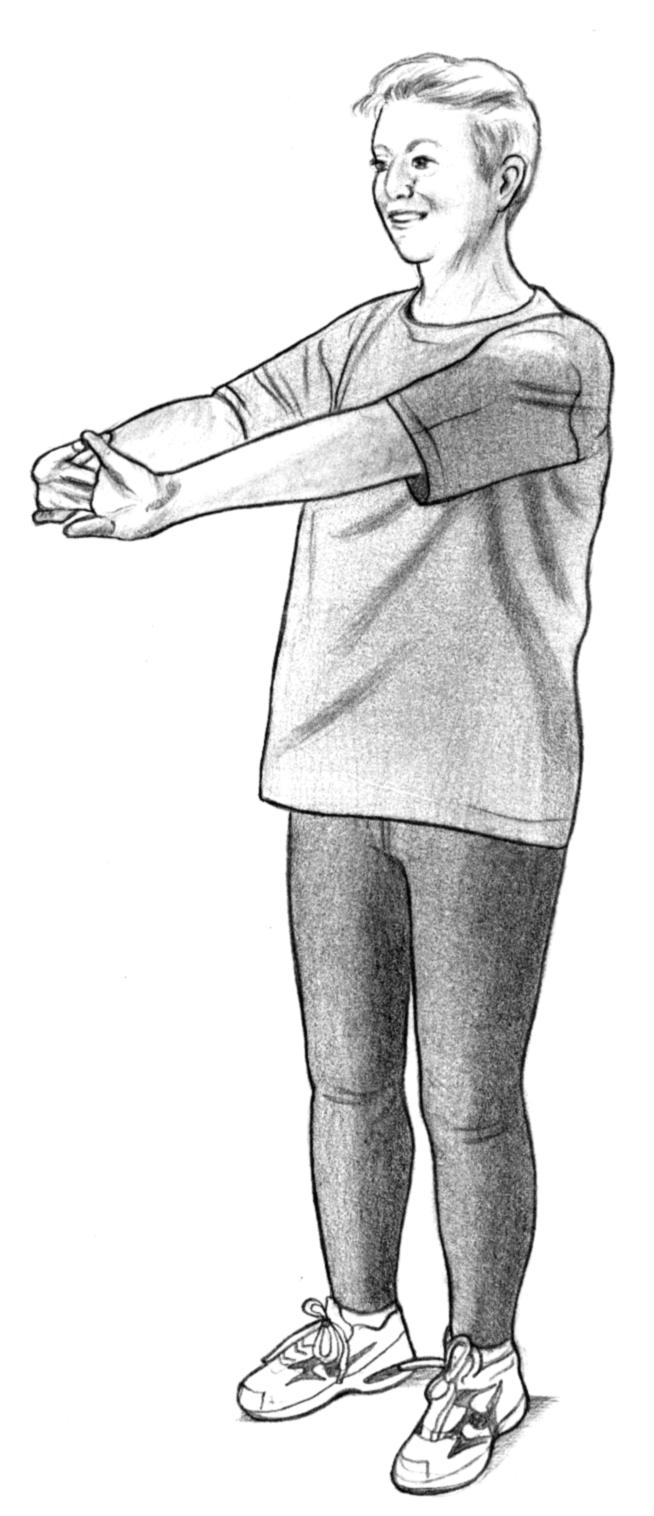


**Balance training module**

**Overview:**

| All participants will receive the balance training begin at Level I.Participants will perform all exercises their current level.Each participant will progress to the next level of balance exercises when all exercises at that level can be performed correctly and without difficulty by the participant. Correctness of performance indicates that the exercisesdemonstrated to the physical activity interventionist by the participant are performed as written in the physicalactivity program, eight out of ten times or 3 out of five times. Difficulty might be indicated if the exercises areperformed with a strained facial grimace, holding one’s breath, or performance of exercises in a jerky, hesitatingmanner.LEVEL I BALANCE EXERCISES | |
| --- | --- |
| The Sink Hip Circle I  1. Stand facing kitchen sink 2. Hold on with both hands 3. Do not move shoulders or feet 4. Make a big circle to left with hips 5. Repeat 5 times 6. Make a big circle to right with hips 7. Repeat 5 times | 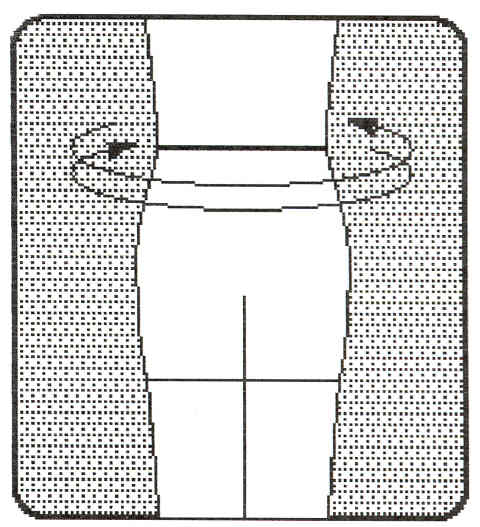  (A) |
|  |  |
| The Sink Toe Stand I  1. Stand facing kitchen sink 2. Hold on with both hands 3. Go up on your toes 4. Hold for count of 5 5. Then come down 6. Repeat 10 times | 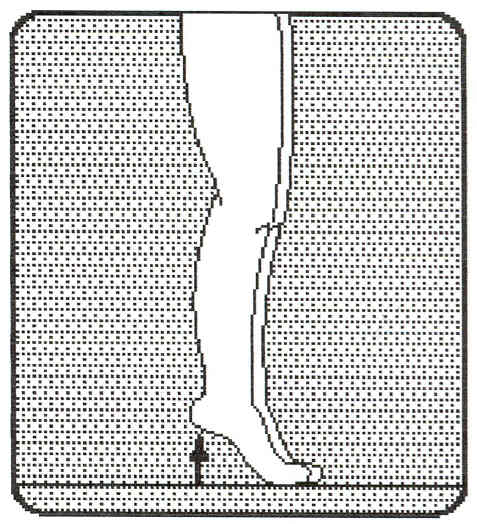  (B) |
|  |  |
| One Leg Sink Stand I  1. Stand facing kitchen sink 2. Hold on with both hands 3. Stand on your left leg for count of 5 4. Stand on your right leg for count of 5 5. Repeat 10 times | 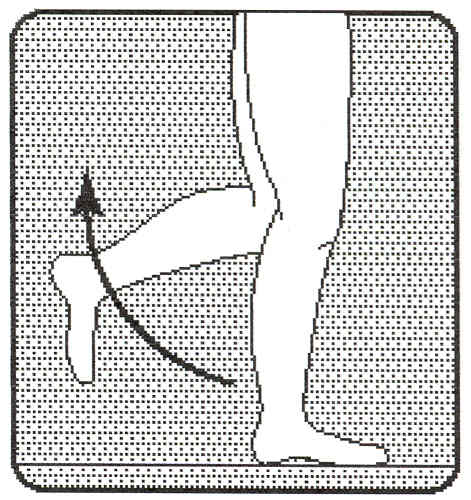  (C) |

|  |  |
| --- | --- |
| Sink Side Step I  1. Stand facing kitchen sink 2. Hold on with both hands 3. Move hands along kitchen sink as you step to left 5 steps 4. Step with both feet to right 5 steps 5. Repeat 5 times | 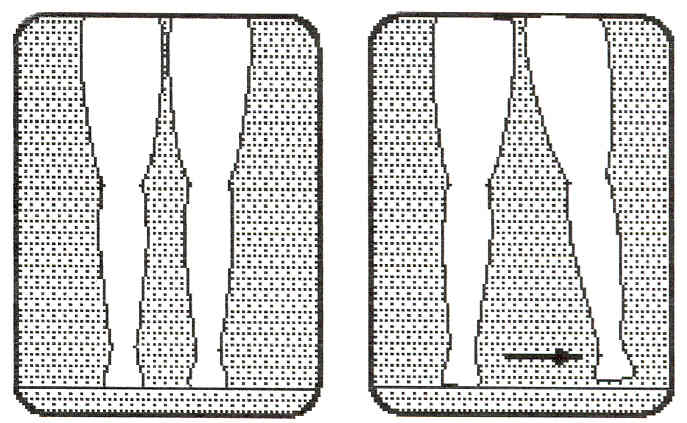  (D) |

| LEVEL II BALANCE EXERCISES | |
| --- | --- |
| The Sink Toe Stand II  1. Stand facing kitchen sink 2. Hold on with one hand 3. Go up on your toes 4. Hold for count of 5 5. Then come down 6. Repeat 10 times | 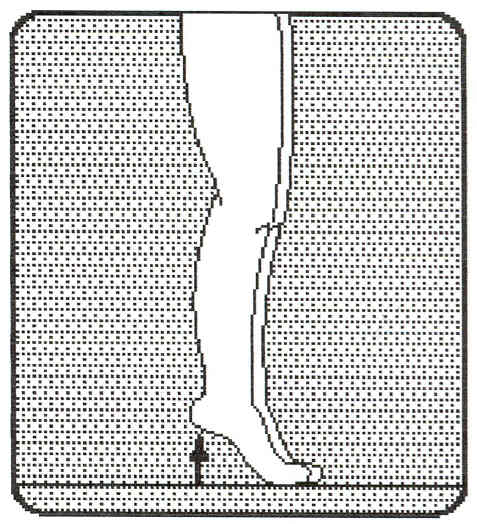  (A) |
|  |  |
| One Leg Sink Stand II  1. Stand facing kitchen sink 2. Hold on with both hands 3. Stand on your left leg for count of 5 4. Stand on your right leg for count of 5 5. Repeat 10 times | 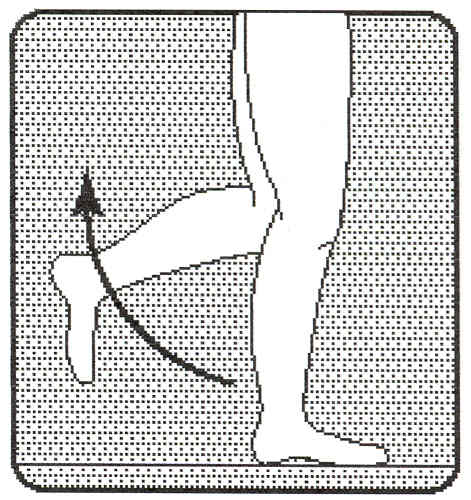  (B) |
|  |  |
| Sink Side Step II  1. Stand facing kitchen sink 2. Hold on with one hand 3. Move hand along kitchen sink as you step to left 5 steps 4. Step to right 5 steps 5. Repeat 5 times | 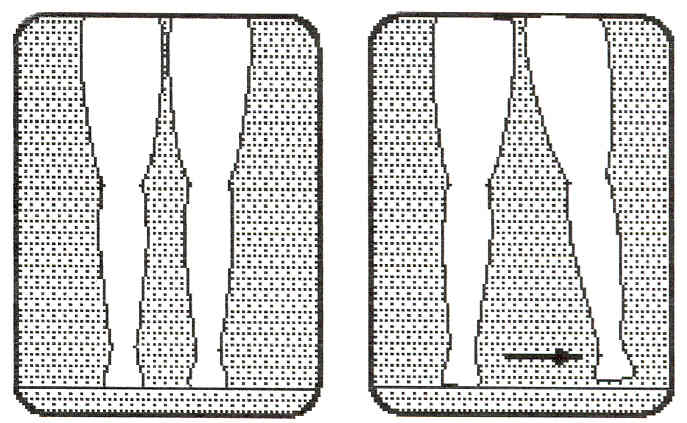  (C) |

| Step forward II  1. Stand with right side toward kitchen sink. 2. Hold onto the sink with your right hand. 3. Step forward with your left leg; shift your weight forward over the left leg. 4. Do not step forward with the right leg. 5. Return the left leg to the starting position. 6. Repeat on opposite side. 7. Continue to alternate each leg. 8. Repeat 10 times. |  |
| --- | --- |
|  |  |
| **Step backward II**  1. Stand with right side toward kitchen sink. 2. Hold onto the sink with your right hand. 3. Step backward with your left leg; shift your weight backward over the left leg. 4. Do not step backward with the right leg. 5. Return the left leg to the starting position. 6. Repeat on opposite leg. 7. Continue to alternate each leg. 8. Repeat 10 times. |  |

| LEVEL III BALANCE EXERCISES | | |
| --- | --- | --- |
|  |  | |
| Sink Leg Cross III  1. Stand facing kitchen sink 2. Hold on with both hands 3. Move hands along kitchen sink as you step 4. Cross foot in front of right foot 5. Take a side step with your right foot passing it out from behind your left foot 6. Repeat steps 4 & 5 three times 7. Now, cross right foot in front of left foot (reverse directions) 8. Take a side step with your left foot passing it out from behind your right foot 9. Repeat steps 7 & 8 three times | |   (A) |
|  |  | |
| Sink Side Step III  1. Stand facing kitchen sink 2. Do not hold onto sink 3. Step to left 5 steps 4. Step to right 5 steps 5. Repeat 5 times | 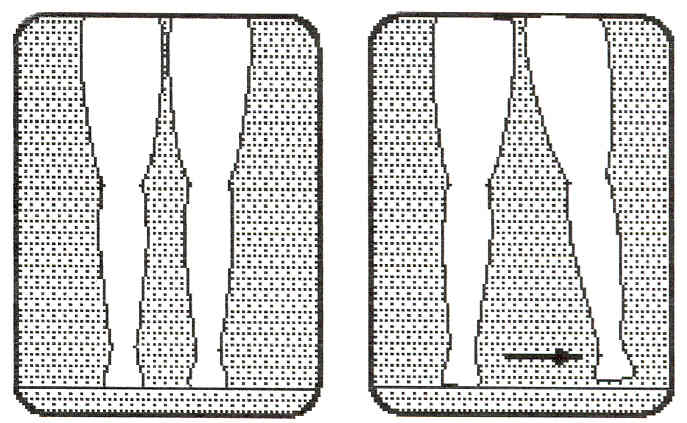  (B) | |

|  |  |
| --- | --- |
| The Sink Toe Stand III  1. Stand facing kitchen sink 2. Do not hold onto the sink 3. Go up on your toes 4. Hold for count of 5 5. Then come down 6. Repeat 10 times | 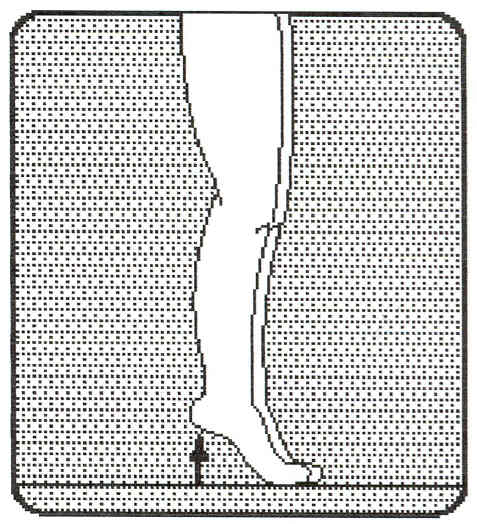  (E) |

| Step forward III 1.Stand with right side toward kitchen sink.   1. Balance with fingertips of the right hand. 2. Step forward with your left leg; shift your weight forward over the left leg. 3. Do not step forward with the right leg. 4. Return the left leg to the starting position. 5. Repeat on opposite side. 6. Continue to alternate each leg. 7. Repeat 10 times. |  |
| --- | --- |
|  |  |
| **Step backward III**  1. Stand with right side toward kitchen sink. 2. Balance with fingertips of the right hand. 3. Step backward with your left leg; shift your weight backward over the left leg. 4. Do not step backward with the right leg. 5. Return the left leg to the starting position. 6. Repeat on opposite leg. 7. Continue to alternate each leg. 8. Repeat 10 times. |  |

| LEVEL IV BALANCE EXERCISES | |
| --- | --- |
| One Leg Sink Stand IV  1. Stand facing kitchen sink 2. Do not hold onto the kitchen sink 3. Stand on your left leg for count of 5 4. Stand on your right leg for count of 5 5. Do each leg 10 times |   (A) |
|  |  |
| Tandem Walking IV  1. Stand with left side toward kitchen sink 2. Hold on with left hand 3. Move hand along kitchen sink as you step 4. Place right heel directly in front of toes of right foot 5. Now place left heel directly in front of toes of right foot 6. Repeat steps 4 and 5 three times 7. Turn around 8. Hold with right hand 9. Repeat steps 4 and 5 three times |   (B) |

| Cross-over Walk IV  1. Stand with left side toward kitchen sink 2. Hold on with left hand 3. Move hand along kitchen sink as you step forward 4. Cross left foot over right foot 5. Cross right foot left foot 6. Repeat steps 4 and 5 three times 7. Turn around 8. Hold with right hand 9. Repeat steps 4 and 5 three times |   (C) | |
| --- | --- | --- |
|  |  | |
| **Step Backward and Forward IV**  1. Stand with right side toward kitchen sink. 2. Hold onto the sink with your right hand. 3. Step backward with your left leg, shift your weight backward over the left leg, pause. 4. Step forward with the left leg, past the right leg. 5. Shift your weight forward over the left leg. 6. Return the left leg to the starting position. 7. Repeat on opposite leg. 8. Continue to alternate each leg. 9. Repeat 10 times. | |  |
|  |  | |

| **Modified Step-up IV**   1. Stand with your right side toward the sink. 2. Hold on to the sink with your right hand. 3. Have a step stool or large book (phone book) on the floor in front of you. 4. Lift the right foot up and tap it on the stool/book. 5. Lower the right foot back to the floor. 6. Repeat with the left foot. 7. Alternate right and left steps. 8. Repeat 10 times. |  |
| --- | --- |

| LEVEL V BALANCE EXERCISES | |
| --- | --- |
| Walk with Head Turns V  1. Stand with right side toward sink. 2. Hold on with the right hand. 3. Move hand along sink as you step. 4. Take 5 steps forward. 5. While stepping forward turn your head to the right and then to the left one time. 6. Turn around. 7. Hold with the left hand. 8. Repeat steps 4 and 5. 9. Repeat entire sequence 5 times. |  |
|  |  |
| Forward Lunge V  1. Stand with side to kitchen sink 2. Hands on hip 3. Lunge forward with right foot 4. Return to start position 5. Lunge forward with left foot 6. Return to start position 7. Repeat 10 times | 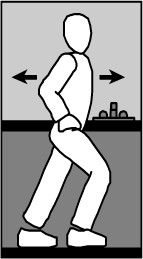 |

| Side Lunge, Hands on Hips V  1. Stand near kitchen sink 2. Hands on hip 3. Lunge to side on left foot 4. Return to upright position 5. Lunge to side on right 6. Return to upright position 7. Repeat 10 times 8. Repeat 10 times |  | | |
| --- | --- | --- | --- |
|  |  | | |
| **Step Backward and Forward V**  1. Stand with right side toward kitchen sink. 2. Balance with fingertips of your right hand. 3. Step backward with your left leg, shift your weight backward over the left leg, pause. 4. Step forward with the left leg, past the right leg. 5. Shift your weight forward over the left leg. 6. Return the left leg to the starting position. 7. Repeat on opposite leg. 8. Continue to alternate each leg. 9. Repeat 10 times. | | |  |
|  |  | | |
| **Modified Step-up V**  1. Stand with your right side toward the sink. 2. Balance with fingertips of your right hand. 3. Have a step stool or large book (phone book) on the floor in front of you. 4. Lift the right foot up and tap it on the stool/book. 5. Lower the right foot back to the floor. 6. Repeat with the left foot. 7. Alternate right and left steps. 8. Repeat 10 times. | |  | |

**Potential Illnesses and Injuries: Sorting out the Expected from the Unexpected**

The following table lists the types of health complaints that participants tend to mention over the telephone. The health issues in the left-hand column are ordinary/common and generally are not cause for great concern. The issues in the right-hand column are atypical and warrant more attention. If you hear a participant mention issues in the right-hand column, please contact project manager so that you and the intervention team can develop an appropriate and safe action plan for the participant.

| **Common:** | **Uncommon:** |
| --- | --- |
| - **Muscle soreness** - **Stiffness** - **Runny nose** - **Allergies (without chest congestion or asthma)** - **Stress** - **Fatigue** | - **Pulled or strained muscles, tendons, or ligaments.** - **Joint pain** - **Joint swelling** - **Ankle or knee injury** - **Chronic foot pain** - **Back pain** - **Neck pain** - **Decreased range of motion in joint** - **Broken bones** - **Recurring blisters** - **Fever** - **Chest congestion or chest cough** - **Asthma** - **Flu** - **Bronchitis** - **Pneumonia** - **Surgery** - **Other illness** |

**Keys to Counseling during Difficult Situations:**

1. Be nonjudgmental
2. Be empathic
3. Give individualized advice to help problem solve
4. Don’t ask “why” or close-ended questions
5. Don’t take ownership of the participant’s behavior
6. Don’t interpret (when a paraphrase will do)
7. Stick to the “Here and Now”
8. Stick with the topic of exercise
9. Ask for help when you need it
10. Give encouragement and praise for the small things

You are encouraged to seek support from your supervisory staff at any time. Seek consultation when:

1. You have a question about exercise, or when you can’t answer a question posed by your participant.
2. You want to validate or verify something you told your participant.
3. You have a particular difficulty with your participant. For example, if he or she talks too much, or is resistant to your suggestions, and you have difficulty finding a solution, seek consultation from an objective third person.

Seek consultation ***immediately*** if a participant talks about depression, alcohol, drugs, abuse of self or another, or suicide.

**Intervention Safety:**

**Overview:**

Safety screening for morbid conditions, protection against potential risks, safety precautions and the data safety monitoring plan are all components of participant safety. For the intervention we have developed specific criteria and protocols for suspending or stopping physical activity and have developed a protocol to adjust the program for intercurrent illness. We have developed specific documentation forms reporting adverse events that occur during the interventions. Subjects will also be instructed on self-monitoring of signs and symptoms of possible emergent conditions.

During the physical activity sessions an automated external defibrillator (AED) is available. All interventionists will be CPR and AED certified. Also, institutional and community EMS services will be activated if needed. During each center-based visit, subjects will sign an attendance sheet and log any health-related problems or symptoms they are experiencing. These sheets will be reviewed by intervention staff before physical activity’s initiated. As indicated previously, participants will be taught the importance and proper method of warming-up prior to and cooling-down following structured activity sessions. If at any point during a physical activity session, participants develop chest pain (including chest discomfort or pressure, left arm pain, or report indigestion or stomach discomfort), shortness of breath, or dizziness, they will be instructed to rest and to contact the center and their physicians if these symptoms persist or recur with further exercise. During the training sessions, we will monitor blood pressure, heart rate and symptoms to identify participants who may show abnormal responses to physical activity (i.e., decrease in systolic blood pressure (≥20 mm Hg); increase in systolic blood pressure to ≥250 mm Hg or in diastolic blood pressure ≥115 mm Hg; frequent premature ventricular contractions; a resting heart rate >120 bpm or increase in heart rate ≥90% of age predicted maximum). These individuals will be instructed to see their physicians before continuing with the physical activity program. If exaggerated blood pressures, heart rate, or symptoms develop during center-based physical activity sessions, the intervention staff should also contact the study physician and/or the participant’s PCP. Currently blood pressure and heart rate are monitored before and after the walking activity at each center-based intervention session. To enhance safety, blood pressure and heart rate are monitored during the walking in participants who had experienced any of the following at a previous physical activity session:

- Resting blood pressure systolic > 200 mm Hg or diastolic > 100 mm Hg
- Decrease in systolic blood pressure ≥20 mm Hg following the activity
- Increase in systolic blood pressure to ≥250 mm Hg or in diastolic blood pressure ≥115 mm Hg following the activity
- Resting heart rate >120 bts./min or < 45 bts./min
- Increase in heart rate ≥90% of age predicted maximum
- Unusual or severe shortness of breath
- Chest pain including chest discomfort or pressure, left arm pain, report of indigestion, or stomach discomfort
- Palpitations
- Light headedness, dizziness or feeling about to faint

A physical activity session had to be discontinued because of other symptoms excluding musculoskeletal symptoms (e.g., knees, ankles, hips) reported by the participant.

*If participants exhibit hypertensive responses, exaggerated heart rate responses, or symptoms with exercise, they will be referred to their PCP for follow up and blood pressure and heart rate will be subsequently monitored during exercise.

Use of proper footwear during physical activity is essential for all participants. The use of silica gel or air midsoles is to be encouraged, as is the use of polyester or cotton-polyester blend socks to keep the feet dry and prevent blisters. Participants should also be taught to inspect their feet for blisters and other damage daily. For participants with severe peripheral neuropathy, or the presence of a foot ulcer, non-weight-bearing physical activity’s recommended, such as swimming and bicycling. For participants with severe neuropathy, walking for more than 30 minutes should be avoided.
